# Self‐Sustaining Hybrid Passive Cooler Enabling Highly Effective Thermal Management for Outdoor Electronics

**DOI:** 10.1002/advs.77183

**Published:** 2026-08-14

**Authors:** Qingyuan Du, Meng Yang, Maoning Li, Guangzhe Chen, Yunpeng Hu, Dandan Li, Dazhi Sun

**Affiliations:** ^1^ State Key Laboratory of Quantum Functional Materials, Guangdong Provincial Key Laboratory of Functional Oxide Materials and Devices, Department of Materials Science and Engineering Southern University of Science and Technology Shenzhen Guangdong China

**Keywords:** atmospheric moisture harvest, hybrid passive cooler, latent heat evaporation, radiative cooling, sensible heat absorption

## Abstract

Outdoor electronics serve as fundamental infrastructure in modern society, yet their reliable operation is critically challenged by simultaneous intense solar radiation and high‐power thermal shocks. To address this challenge, we proposed an innovative hybrid passive cooler (HPC) that integrates a porous vapor‐permeable radiative cooling coating and an autonomous atmospheric moisture‐harvesting hydrogel within a melamine sponge skeleton. A synergistic interplay of radiative cooling, sensible‐heat absorption, and latent‐heat evaporation is thus realized for exceptional outdoor electronics thermal management. Under continuous solar irradiation, the HPC enabled the heater to maintain a temperature of averaging 8.3°C below ambient air and achieve a maximum temperature drop of ∼42.4°C when further subjected to an intense thermal shock of 2000 W·m^−2^ for 30 min. Notably, by leveraging its autonomous atmospheric moisture‐harvesting capability, the HPC could consistently deliver stable and reliable thermal management performance for about 3 h with combined high thermal shock and solar radiation. Therefore, this work provides an efficient, sustainable, and scalable thermal management solution for outdoor electronics.

## Graphical abstract

An innovative hybrid passive cooler integrates a porous radiative coating and an atmospheric moisture‐harvesting hydrogel within a melamine sponge skeleton, synergizing radiative cooling, sensible‐heat absorption, and latent‐heat evaporation. It maintains an average 8.3°C sub‐ambient cooling under sunlight and a maximum ∼42.4°C drop under 2000 W·m^−^^2^ thermal shock, and further delivering stable performance for about 3 hours. This work offers an efficient and sustainable thermal management solution for outdoor electronics.

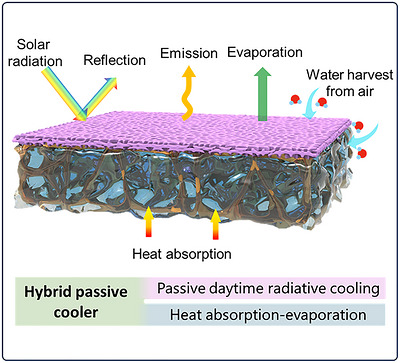

## 1 Introduction

With the rapid development of modern society, outdoor electronics—such as 5G base stations, Internet of Things sensors, and photovoltaic inverters—have become critical pillars supporting today's social operations [1, 2, 3, 4]. Nevertheless, these devices face two major thermal management challenges under prolonged outdoor service [5, 6, 7]. On one hand, intense solar radiation, with an incident intensity of over 1000 W·m^−^^2^ at noon, continuously elevates the surface temperatures of outdoor electronics, thereby inducing internal overheating that degrades performance and potentially causes permanent damage [8, 9, 10]. On the other hand, high‐power operation of outdoor electronics frequently induces severe thermal shocks, characterized by sudden temperature spikes that compromise the stability and service life of electronic components [11, 12, 13]. The combined effect of heat accumulation and cyclic thermal shock not only degrades component performance, but also raises the likelihood of working system failure [14, 15, 16]. This results in heightened maintenance burdens and risks disruption to critical infrastructure like communications and renewable energy. Consequently, effectively addressing the dual thermal challenges of intense solar radiation and high‐frequency thermal shock in outdoor electronics is imperative for ensuring the reliability and sustainability of outdoor electronics that support modern societal functions [17, 18, 19].

Conventional thermal management strategies for electronics rely heavily on active cooling technologies—such as air conditioners, liquid cooling systems—which demand substantial energy input [20]. However, integrating such systems into outdoor electronics often incurs prohibitively high costs due to installation constraints and energy consumption concerns [21]. In recent years, passive radiative cooling (PRC) materials have emerged as a promising zero‐energy alternative solution for outdoor objects thermal management, leveraging sunlight reflection and mid‐infrared emission to outer space to achieve sub‐ambient cooling [22, 23, 24]. For instance, early studies achieved sub‐ambient cooling for objects by developing materials with high solar reflectance and mid‐infrared emittance, thereby advancing daytime radiative cooling technology [25, 26, 27]. Recent advances have diversified radiative cooling toward practical applications, including transparent cooling for vehicles [28], tunable systems for adaptive thermal management [29, 30, 31, 32], multifunctional cooling‐heating surfaces, and flexible designs for wearable electronics [33, 34]. These developments collectively underscore the growing recognition that passive cooling strategies must evolve beyond single‐mechanism approaches to address complex real‐world thermal challenges. However, the cooling capacity (∼150 W m^−2^) of PRC materials is inherently limited by their optical and thermal properties, restricting further performance improvements [34, 35, 36]. To overcome this limitation, some other researchers have developed hybrid cooling strategies that integrate radiative cooling with latent heat absorption mechanisms [37, 38]. This design combines organic phase change materials with radiative cooling materials, utilizing the latent heat of phase change material for daytime cooling and nighttime thermal storage to improve overall energy efficiency [39]. Although such design has achieved significant advancements compared to traditional PRC materials in outdoor objects thermal management, the relatively low latent heat capacity of organic phase change materials limits their effectiveness in outdoor electronics when subject to high‐power and long‐term thermal shocks [17, 19]. Consequently, another research direction focuses on synergizing radiative cooling with the water evaporation (∼2440 J g^−1^) to achieve enhanced cooling performance [40, 41, 42, 43]. Yet, the practical application of the bilayer systems‐fabricated by simple stacking of radiative cooling and evaporative cooling layers‐is challenging [44, 45, 46]. Critical issues such as inherent layer separation and the impediment of atmospheric moisture harvesting efficiency by the radiative cooling layer have yet to be adequately addressed, making the actual cooling performance difficult to predict and sustain [47, 48, 49, 50]. Overall, while significant progress has been made in hybrid passive cooling technology, existing solutions remain inadequate for ensuring the long‐term reliability and sustainability of outdoor electronics under intense solar radiation and high‐frequency thermal shocks. Furthermore, their complex fabrication poses a formidable barrier to widespread adoption. Therefore, the development of efficient, robust, and integrated passive cooling technologies capable of simultaneously mitigating solar radiation and thermal shocks remains a critical and urgent challenge.

Against this backdrop, there is a pressing need to develop a low‐energy, high‐reliability passive cooler for outdoor electronics that can simultaneously achieve efficiently passive daytime radiative cooling and thermal‐shock resistance during operations. In this work, we propose a self‐sustaining hybrid passive cooler (HPC) constructed by integrating a porous, vapor‐permeable P(VdF‐HFP) radiative cooling coating with an atmospheric moisture‐harvesting Polyacrylamide (PAm) hydrogel into a melamine sponge (MS) matrix, thus forming a combination of structure and function. This integrated design achieves the synergistic interplay of radiative cooling, sensible heat (SH)‐absorption, and latent heat (LH)‐evaporation. The HPC thus not only realizes high passive cooling performance passive radiative cooling through porous P(VdF‐HFP) radiative cooling coating and LH‐evaporation for outdoor electronics, but also mitigate temperature fluctuations during high‐power thermal shock by leveraging the moisture sorption‐desorption cooling capability of the PAm hydrogel, thereby providing a practical solution toward enhancing the long‐term reliability and energy efficiency of outdoor electronics.

## 2 Result and Discussion

### 2.1 Hybrid Passive Cooler Strategy for Outdoor Electronics

**FIGURE 1:**
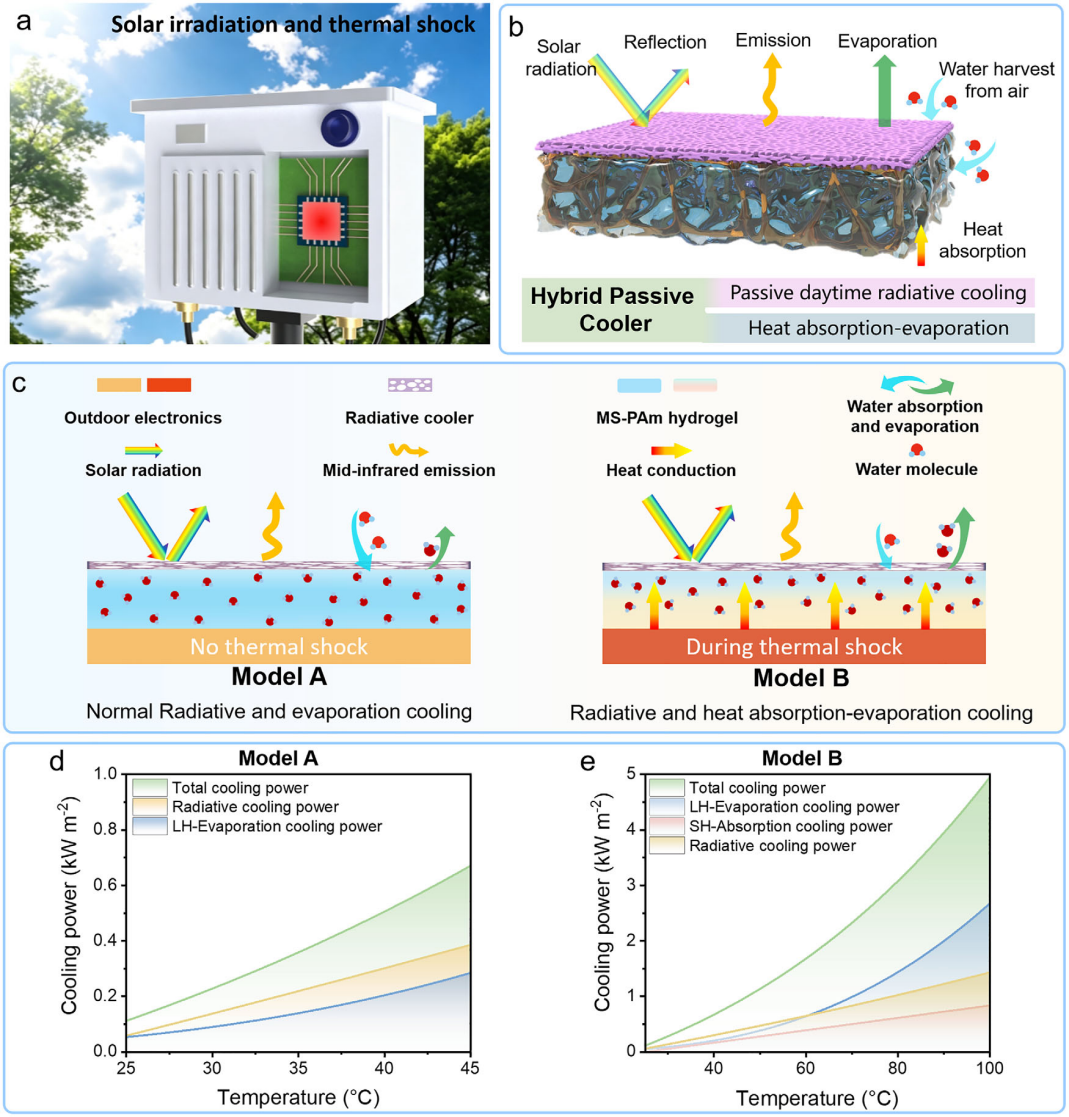
Design of hybrid passive cooler (HPC). (a) Schematic diagram of thermal management issues in outdoor electronics during solar radiation and thermal shock. (b) Schematic diagram of the structure and multi‐mechanism synergistic cooling principle of HPC. (c) Comparison of cooling modes under no thermal shock (Model A) and during thermal shock (Model B). (d) Cooling power calculation of HPC under no thermal shock scenario (25°C–45°C): synergy of radiative cooling and LH‐evaporation cooling. (e) Cooling power calculation of HPC under thermal shock scenario (25°C–100°C): synergy of radiative cooling, SH‐absorption and LH‐evaporation cooling.

Outdoor electronics are critical to modern infrastructure but suffer from thermal challenges caused by solar exposure and operational thermal shocks (Figure 1a, schematic illustration). For 5G base station (Figure S1, where thermal interface materials connect the high‐power chip to the enclosure, transferring heat from the chip to the enclosure finned), a typical example, conventional radiative cooling materials [8] and finned heatsinks [17, 19, 41] become inadequate during high‐power operation (The heat flux far exceeds 1000 W m^−^^2^, Figure S2). To address this limitation, we propose the hybrid passive cooler (HPC) that integrates a porous vapor‐permeable radiative cooling coating and an autonomous atmospheric moisture‐harvesting hydrogel within a melamine sponge skeleton. This design synergistically combines radiative cooling with the sensible heat (SH)‐absorption and latent heat (LH)‐evaporation properties of the hydrogel, significantly improving cooling effect under combined solar radiation and high‐power thermal shock conditions. The HPC integrates a mechanically interlocked P(VdF‐HFP) radiative cooling coating via spraying and an in‐situ formed PAm hydrogel within an MS substrate (Figure 1b). Notably, the porous coating boosts solar reflectance and vapor permeability. To evaluate its performance for outdoor electronics, theoretical cooling power was calculated for two key scenarios: no thermal shock (25‐45°C) and under thermal shock (25‐100°C). The calculation employed a temperature‐dependent evaporation rate function derived from experimental data (Figure SI), and further theoretical details are provided in Supporting Information S1: Section 1.5.

When the HPC is integrated with outdoor electronics under ambient temperature conditions (25°C–45°C), it provides cooling power by radiative cooling and LH‐evaporation (Figure 1c Left) [51]. The total cooling power provided by the HPC is calculated as follows:

(1) $$P_{total}=P_{radiative\;cooling}+P_{LH-Evaporation}$$

As shown in Figure 1d, under ambient conditions without thermal shock, the radiative cooling power provided by the HPC exceeds its LH‐evaporation power for outdoor electronics. This is attributed to the temperature‐limited evaporation rate of the HPC without thermal shock (Figure SIIa), which curtails the LH‐evaporation cooling capacity relative to radiative cooling (Figure S3). Therefore, the radiative cooling contribution of the HPC for outdoor electronics is more than of LH‐evaporation in the absence of thermal shock. Nevertheless, by synergistically combining radiative cooling with LH‐evaporation, the HPC achieves an integrated cooling performance that surpasses single cooling mechanism under ambient conditions.

When outdoor electronics experience a thermal shock (25°C–100°C), the resulting rapid temperature rise subjects the HPC to intense heating, causing its temperature to surge from ambient within a short period. During this transient, in addition to the radiative cooling power, the PAm hydrogel within the HPC contributes to cooling effect through two distinct mechanisms: SH‐absorption when during the temperature rise, and sustained LH‐evaporation (Figure 1c Right) [52]. Therefore, the total cooling power provided by the HPC for outdoor electronics under combined thermal shock and solar radiation can be described by the following equation:

(2) $$P_{total}=P_{radiative\;cooling}+P_{SH-Absorption}+P_{LH-Evaporation}$$

Specifically, the temperature of the HPC integrated with outdoor electronics also rises rapidly during thermal shock. This dynamic heating enhances the evaporation rate within the PAm hydrogel and creates a significant temperature difference, which collectively amplifies cooling power through SH‐absorption and LH‐evaporation. During this transient heating phase, the cooling mechanism undergoes a sequential transition: initially, sensible‐heat absorption dominates as the hydrogel temperature rises, governed by its effective heat capacity (3.87 J·g^−^^1^·°C^−^^1^ for HPC_45_, Figure S15). Subsequently, the exponentially accelerated evaporation rate (Figure SI) intensifies the latent‐heat contribution, which eventually surpasses sensible‐heat absorption. Under continuous thermal shock, the HPC finally reaches a steady‐state temperature, the combined cooling power from both mechanisms reaches its peak point (Figure SIIb). This steady‐state point was selected for theoretical analysis thus allows for a clearer distinction between the sensible heat and latent heat contributions to the total cooling capacity. Thereafter, in the sustained thermal shock stage, cooling power is predominantly provided by LH‐evaporation. Once the HPC reaches thermal equilibrium (dT/dt = 0), SH‐absorption ceases, and the system becomes entirely dependent on the finite latent‐heat reserve. The evaporation rates of the HPC were measured from 25°C–100°C at 30‐minute intervals. The fitted temperature‐dependent curve (Figure SI) was subsequent used to calculate the cooling power. Analysis of the thermal shock response (Figure 1e and Figure S4) demonstrates that the SH‐absorption power increases linearly with temperature, thereby contributing a portion of the total cooling power. Additionally, when the HPC temperature is below approximately 60°C, the LH‐evaporation cooling power remains lower than the radiative cooling power due to the limited evaporation rate. As the temperature increase further, the evaporation rate of the HPC rises substantially, making LH‐evaporation become the dominant cooling power. This analysis confirms that the SH‐absorption and LH‐evaporation effects, enabled by moisture‐harvesting capability of the HPC, collectively provide an effective thermal‐shock resistance.

Based on this synergistic design that integrating optical, thermal and mass transfer principles, the HPC achieves outstanding performance in both sub‐ambient cooling and thermal shock resistance. To quantitatively benchmark against existing technologies, we systematically compared the HPC with representative passive cooling strategies—including radiative cooling materials, radiative cooling integrated with phase change materials, and other hydrogel cooling systems—across five key performance metrics: thermal shock‐shock resistance, sub‐ambient cooling, cooling power, cycling stability, and weather resistance (Tables S1‐S3 and Figure S5) [41]. Above studies systematically elucidate the theoretical cooling contributions of the HPC—from the combined perspectives of radiative cooling, SH‐absorption, and LH‐evaporation—under simultaneous solar radiation and thermal shock, validating a novel hybrid passive cooling strategy. It provides an effective thermal management solution for outdoor electronics, thereby supporting sustainable development.

### 2.2 Morphology and Optical Properties

The HPC was fabricated by first spraying a phase‐separated P(VdF‐HFP) solution onto an MS skeleton to form a radiative cooling coating, which exhibits excellent optical properties and water vapor permeability [25]. An in‐situ polymerized PAm hydrogel was then formed within the MS skeleton, yielding an integrated structure (Figure S6) that merges radiative cooling with dynamic moisture sorption‐desorption. The MS skeleton provides mechanical interlocking, endowing the HPC with superior structural integrity and mechanical strength compared to conventional bilayers (Figures S9 and S10), underscoring its stability and practical viability.

**FIGURE 2:**
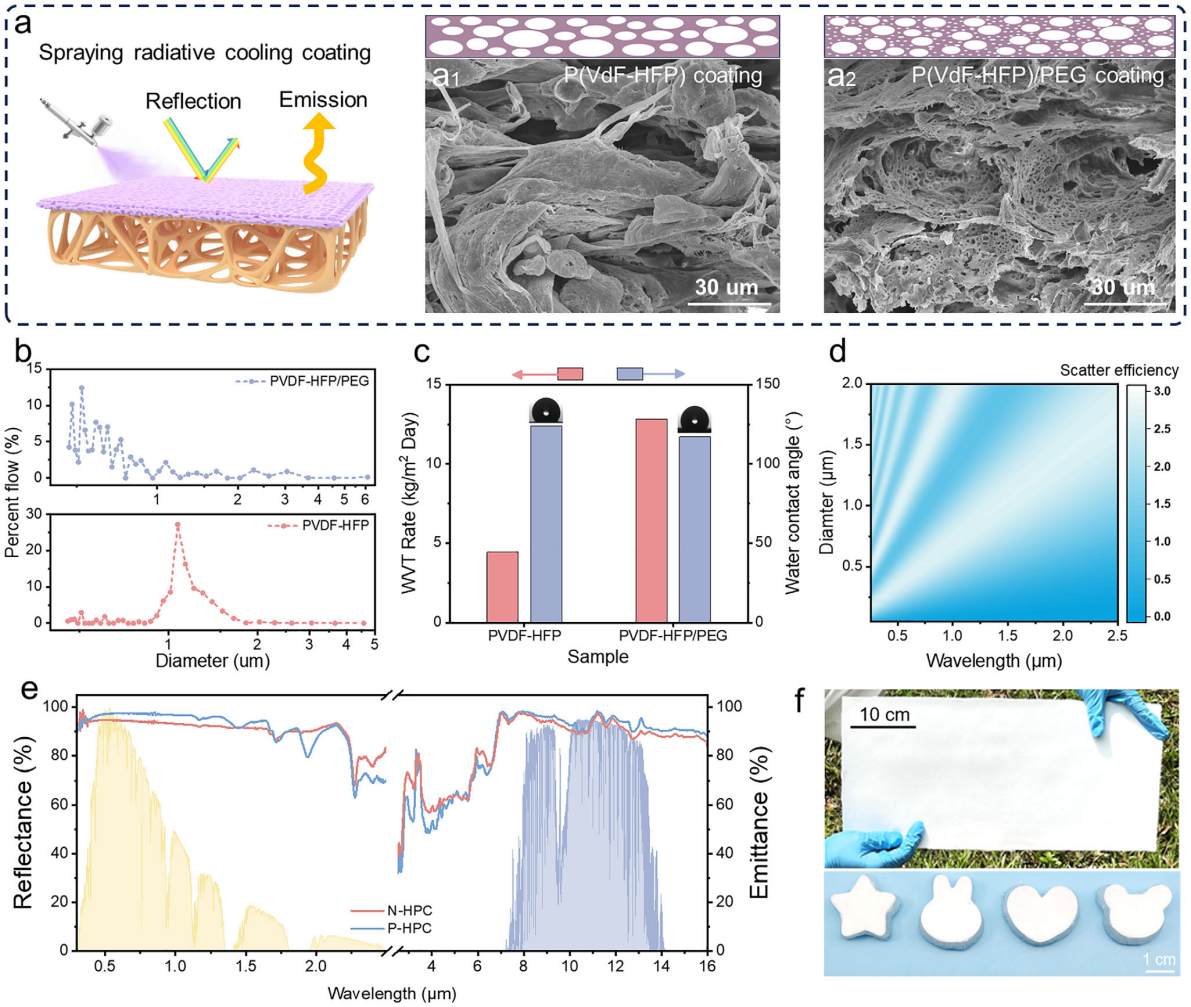
Morphology and optical properties of HPC. (a) Schematic of radiative cooling coating sprayed on MS surface and its microstructure. SEM image of P(VdF‐HFP) coating (a1) and P(VdF‐HFP)/PEG coating (a2). (b) Pore size distribution comparison of samples prepared by different spraying processes. (c) Comparison of WVT rate and water contact angle between P(VdF‐HFP) and P(VdF‐HFP)/PEG coatings. (d) Scattering efficiency of P(VdF‐HFP) with different pore sizes in the wavelength range of 0.25–2.5 µm. (e) Comparison of solar reflectance and infrared emittance spectra for different HPC sample. (f) Outdoor display of large‐scale HPC sample and multi‐shaped cut samples.

The detailed preparation of the P(VdF‐HFP) radiative coating is illustrated in Figure 2a and Figure S7. To optimize pore formation during phase separation, soluble polyethylene glycol (PEG) was introduced as a pore‐forming agent into the aqueous phase, and its effects on the coating's porous morphology and optical properties were investigated. SEM images (Figure 2a_1_,a_2_ and Figure S7) confirm that PEG incorporation yields abundant, uniformly distributed micro‐nano pores. Pore size distribution analysis (Figure 2b) further demonstrates that the PEG‐modified P(VdF‐HFP) coating exhibits a broader size distribution and enhanced connectivity compared to the normal coating with concentrated pores. Moreover, water vapor transmission rate tests (Figure 2c) indicate that the PEG‐modified P(VdF‐HFP) coating achieves higher vapor permeability at the same thickness, providing an efficient transport pathway for water vapor. This hierarchical porous architecture enables atmospheric moisture to permeate through the radiative coating and be captured by the underlying LiCl‐doped PAm hydrogel, while simultaneously maintaining high solar reflectance for radiative cooling, thereby achieving structural and functional synergy between the two components. The P(VdF‐HFP) coating exhibits excellent hydrophobic properties in outdoor applications, and the P(VdF‐HFP) coating of the HPC still maintains good hydrophobicity even after a 100‐day cycle (Figure S8). The resulting porous structure not only significantly enhances water vapor transport but also effectively reduces solar radiation heat by improving sunlight scattering and further mitigates the impact of rainwater on free LiCl inside the HPC.

Finite‐difference time‐domain (FDTD) simulations were performed to evaluate the scattering efficiency of P(VdF‐HFP) materials with pore diameters ranging from 0.1 to 2 µm (Figure S11). As shown in Figure 2d, the micro‐nano porous P(VdF‐HFP) with hierarchical structure effectively scatters visible and near‐infrared light, thereby achieving high reflectivity across the solar spectrum. Figure 2e shows the reflectance and emissivity spectra of conventional HPC (N‐HPC) and porous HPC (P‐HPC) prepared using different processes (see Supporting Information S1:Section 1.3 for details). The average solar reflectivity of P‐HPC and N‐HPC are 0.96 and 0.94, respectively. The significant improvement in P‐HPC is attributed to the hierarchical micro‐nano porous structure induced by PEG, which effectively enhances light scattering. Furthermore, both N‐HPC and P‐HPC exhibit excellent radiative cooling performance, with average emissivity of 0.93 and 0.95, respectively, within the atmospheric transparency window. After 100 days of outdoor cycling tests, the solar reflectance and mid‐infrared emissivity of HPC remained at 0.91 and 0.91 (Figure S12), respectively, indicating that its radiative cooling performance did not show significant degradation during prolonged outdoor cycling. Subsequently, the HPC demonstrates significant application advantages (Figure 2f), with inherent potential for scalability and shape customization to meet diverse practical requirements. Furthermore, the two‐step preparation process (spraying and in‐situ polymerization) employs standard chemical procedures, can be naturally scaled up with sample size, ensures performance consistency across different scales, and can be adapted for industrial production through simple engineering adjustments. This study successfully fabricated a structurally integrated HPC for synergistic radiative cooling and moisture sorption‐desorption. The innovation of using PEG as a pore‐forming agent yielded a coating with high solar reflectivity and vapor permeability, resulting in a mechanically robust device that represents an ideal thermal management solution for outdoor electronics.

### 2.3 Atmospheric Moisture Sorption‐Desorption Properties

**FIGURE 3:**
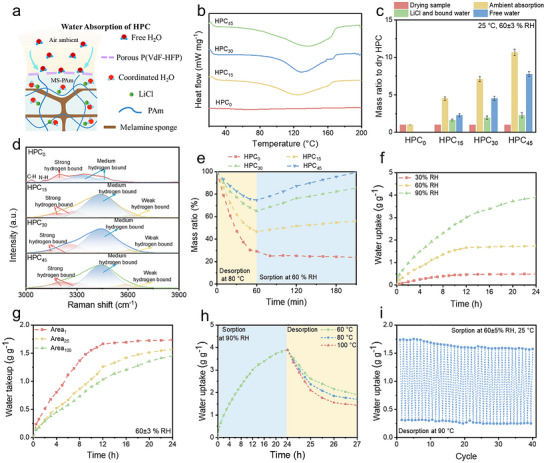
Water vapor sorption‐desorption and thermal performance of HPC. (a) Schematic of water sorption mechanism of HPC. (b) DSC curves of different HPC samples after 7 days environmental exposure. (c) Mass percentage of each component of HPC samples with different concentration of LiCl solution after 7 days environmental exposure. (d) Raman spectra of different HPC samples. (e) Mass ratio variation of different HPC samples during desorption at 80°C and sorption at room temperature and60% RH. (f) Water absorption rate over time for the HPC_45_ sample under varying humidity conditions. (g) Water sorption of the HPC_45_ sample with different surface areas at 60 ± 3% RH. (h) Water desorption of the HPC_45_ sample after sorption at 90% RH under different heating temperatures. (i) Forty water sorption–desorption cycling tests carried out at 25°C, 90% RH for sorption and 90°C for desorption.

For the HPC, the sustained LH‐evaporation cooling is highly dependent on the spontaneous atmospheric moisture harvesting capacity of the PAm hydrogel. Therefore, we further investigate the moisture harvesting capacity of the PAm hydrogel in different humidity conditions. And in order to enhance the dynamic moisture harvesting capacity of the PAm hydrogel, we incorporated LiCl salt via a solution adsorption method.The PAm hydrogel within the HPC was directly immersed into a LiCl solution (Figure S6). This allows LiCl ions to coordinate with the PAm network and water molecules, thereby stably embedding them into the structure and endowing the HPC with the dynamic moisture harvesting capacity under ambient conditions. As illustrated in Figure 3a, environment water vapor that permeates the porous P(VdF‐HFP) coating, is captured by LiCl ions in the HPC, and ultimately converted to coordinated water within the PAm network. This integration of structural, mass‐transport, and optical functions enables efficient atmospheric moisture harvesting. Figure S13 shows DSC results for samples tested immediately after LiCl solution immersion, which exhibit higher enthalpy values in the fully saturated state. Yet for practical application, performance under natural environmental conditions is paramount. For comparison, Figure 3b subsequently presents the DSC curves for HPC samples with varying LiCl loadings (HPC_0_, HPC_15_, HPC_30_, and HPC_45_) after 7 days of ambient exposure. The corresponding phase change enthalpies after spontaneous atmospheric moisture absorption are 237.2, 1129.8, 1638.6, and 1859.0 J g^−1^, respectively. Therefore, a higher concentration of LiCl solution in the HPC can achieve greater cooling capacity from the atmosphere. Furthermore, we employed a gravimetric method to analyze the proportions of free water and LiCl‐bound water in HPC samples, both post‐immersion and after ambient exposure (Figure 3c and Figures S14 and S15, Supporting Information S1: Section 1.4). The results show that the HPC after immersion in higher LiCl concentration solution will lead the bound water increased. The influence of LiCl loading is further investigated by TGA (Figure S16). Notably, HPC_45_ demonstrates the strongest free water absorption capacity under ambient moisture conditions. Correspondingly, its specific heat capacity reaches 3.87 J g^−1^°C^−1^ (Figure S17), significantly exceeding that of other samples. Raman spectroscopy (Figure 3d) further confirms that LiCl concentration effectively modulates the binding state of water molecules, which underpins the enhanced environmental moisture absorption capability.

A critical functional feature of the HPC is its dynamic water vapor sorption‐desorption behavior. Hence, our systematic examining samples with varying LiCl loadings after saturation (Figure 3e). HPC_45_ showed the smallest mass loss and fastest recovery rate during both thermal desorption and subsequent natural re‐sorption. This enhancement phenomenon stems from the high LiCl loading, which effectively regulates the internal water evaporation rate—consistent with DSC analysis—while preserving rapid moisture uptake at ambient temperature. To further assess practical performance and inform cycling strategies, moisture sorption was measured under controlled humidity (30%, 60%, and 90% RH) using an environmental chamber (Figure 3f and Figures S18 and S19). HPC_45_ consistently demonstrated superior sorption capacity across all conditions. Furthermore, after a 100‐day outdoor cycle, the HPC samples still exhibited stable water uptake capacity. Another critical practical factor is the effect of scale on sorption kinetics, that is whether the excellent moisture harvesting performance is retained at larger, application‐relevant dimensions. Tests on HPC_45_ samples with varied surface areas (Figure 3g) confirm that increasing the area over orders of magnitude does not markedly reduce the environmental water collection capacity, affirming its scalability. Subsequent desorption studies on samples pre‐adsorbed at 90% RH and heated to 60°C, 80°C, and 100°C (Figure 3h) show that higher temperatures accelerate desorption rates and increase the total mass released. This pronounced temperature dependence indicates that the HPC can deliver efficient cooling via rapid water release during high‐temperature thermal shocks. Finally, after 40 consecutive sorption (25°C, 60±5% RH)–desorption (90°C) cycles (Figure 3i), HPC_45_ exhibited no significant degradation in water collection capacity, demonstrating exceptional cycling stability for long‐term deployment. Above studies demonstrates not only excellent thermal response and cyclic durability but also the capacity for dynamic atmospheric water harvesting, which translates directly into usable cooling power.

### 2.4 Thermal Shock Resistance Performance

Outdoor electronics under high‐power operation face intense thermal shocks reaching 2000–3000 W m^−^^2^, causing dangerous temperature excursions. To assess HPC cooling performance under thermal shock conditions, we simulated the process using custom setups with commercial heaters (whose spectral characteristics are provided in Figure S20) as controllable heat sources. Temperature profiles of a bare heater, a TPC (Traditional passive cooler, the preparation process is described in Supporting Information Section 1.3)‐integrated heater, and an HPC‐integrated heater were compared.

**FIGURE 4:**
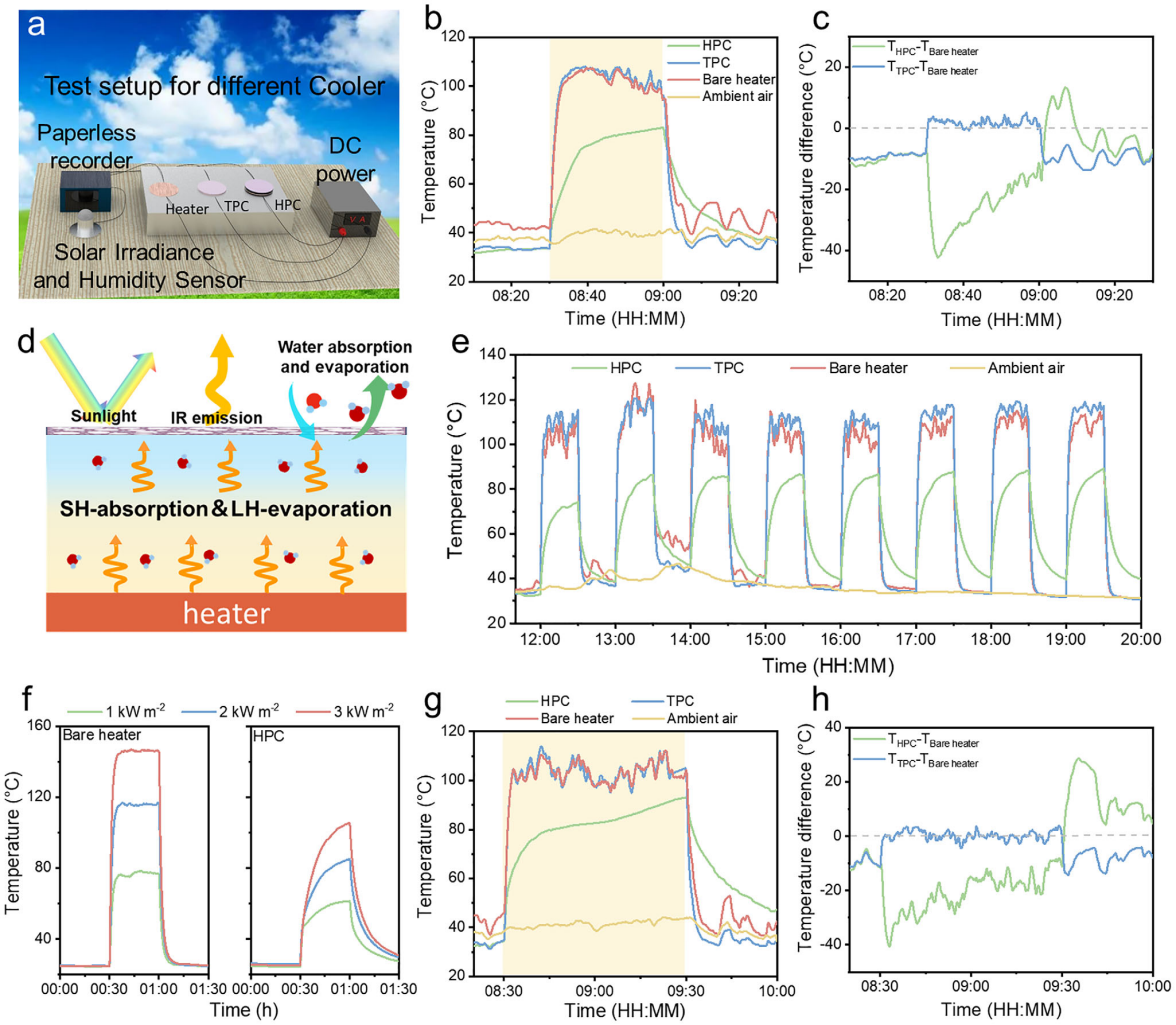
Thermal shock performance of HPC. (a) Schematic diagram of outdoor test setup for different coolers. Temperature variations (b) and temperature difference (c) of different coolers under 2000 W m^−2^ thermal‐shock and solar radiation with 30 min duration. (d) Schematic diagram of the heat transfer model for HPC during the heater thermal shock process. (e) Temperature variations of different coolers during an 8‐interval, 30‐minute‐period of 2000 W m^−^^2^ thermal shock process from 12:00 PM to 8:00 PM. (f) Comparison of temperature variations of the bare heart and the HPC covered one under different thermal‐shock power density. Temperature variations (g) and temperature difference (h) of different coolers under 2000 W m^−2^ thermal‐shock and solar radiation with one hour duration.

A self‐built test setup (Figure 4a and Figure S21) was employed to compare the temperature responses under three cooling strategies: bare heater, heater with the TPC, and heater with the HPC. First, under equal‐area cooler conditions, temperature variations during a continuous 30‐minute thermal shock at 2000 W m^−2^ were monitored (Figure 4b,c and Figure S22). The results show that both the bare heater and the TPC‐integrated heater exhibited rapid temperature surges, eventually reaching very high levels. Notably, although exhibiting effective radiative cooling, the TPC‐integrated heater ultimately surpassed the bare heater in temperature under high‐power thermal shock due to its suppression of convective dissipation, which collectively preclude effective thermal‐shock resistance. This highlights the fundamental limitation of traditional passive materials under such extreme conditions. In contrast, the HPC‐integrated heater showed a markedly suppressed temperature rise, achieving a maximum temperature drop of 42.4°C and an average cooling effect of 26.3°C in 30 min. Comparative experiments further show TPC, pure hydrogel, and HPC achieved average reductions of −1.8°C, 20.7°C, and 27.2°C for heater, respectively (Figure S23). The 6.5°C performance gap between HPC and pure hydrogel quantitatively demonstrates the synergistic gain from combining radiative cooling with hydrogel evaporation, underscoring radiative cooling's necessity for sub‐ambient temperature reduction in outdoor electronics. The thermal‐shock resistance mechanism of HPC is illustrated in Figure 4d: upon thermal shock, a substantial portion of heat is first conducted to the PAm hydrogel, providing immediate cooling via SH‐absorption. Simultaneously, the water evaporation of the HPC accelerates, carrying away substantial heat through LH‐evaporation. Through the dual mechanism of SH‐absorption and LH‐evaporation, the HPC achieves effective thermal‐shock resistance. It should be noted that the moisture sorption rate of the HPC is lower than its evaporation rate during thermal shock. COMSOL simulations (Figures S24–S26) further validate that the HPC exhibits excellent thermal‐shock resistance. Moreover, multiple cyclic tests (Figure 4e and Figures S27, S28) confirmed that the HPC consistently provided effective protection over eight consecutive 30‐minute tests. The cooling effect was strongest under initial strong midday solar radiation and slightly declined in later cycles, as frequent thermal shocks hindered full moisture recharge from the environment. The thermal‐shock resistance was even more pronounced at higher power densities (Figure 3f and Figure S29), with a maximum temperature drop of 78.6°C at 3000 W·m^−^^2^. To probe the limits, a 2000 W·m^−^^2^ shock was extended to an hour (Figure 4g,h, and Figure S30). The thermal‐shock resistance gradually weakened after 30 min, primarily due to moisture depletion which stabilized the HPC temperature and reduced the evaporation rate. This insight directly informs the design of HPC systems targeting long‐duration, high‐power thermal management, pointing to a critical path for future development.

**FIGURE 5:**
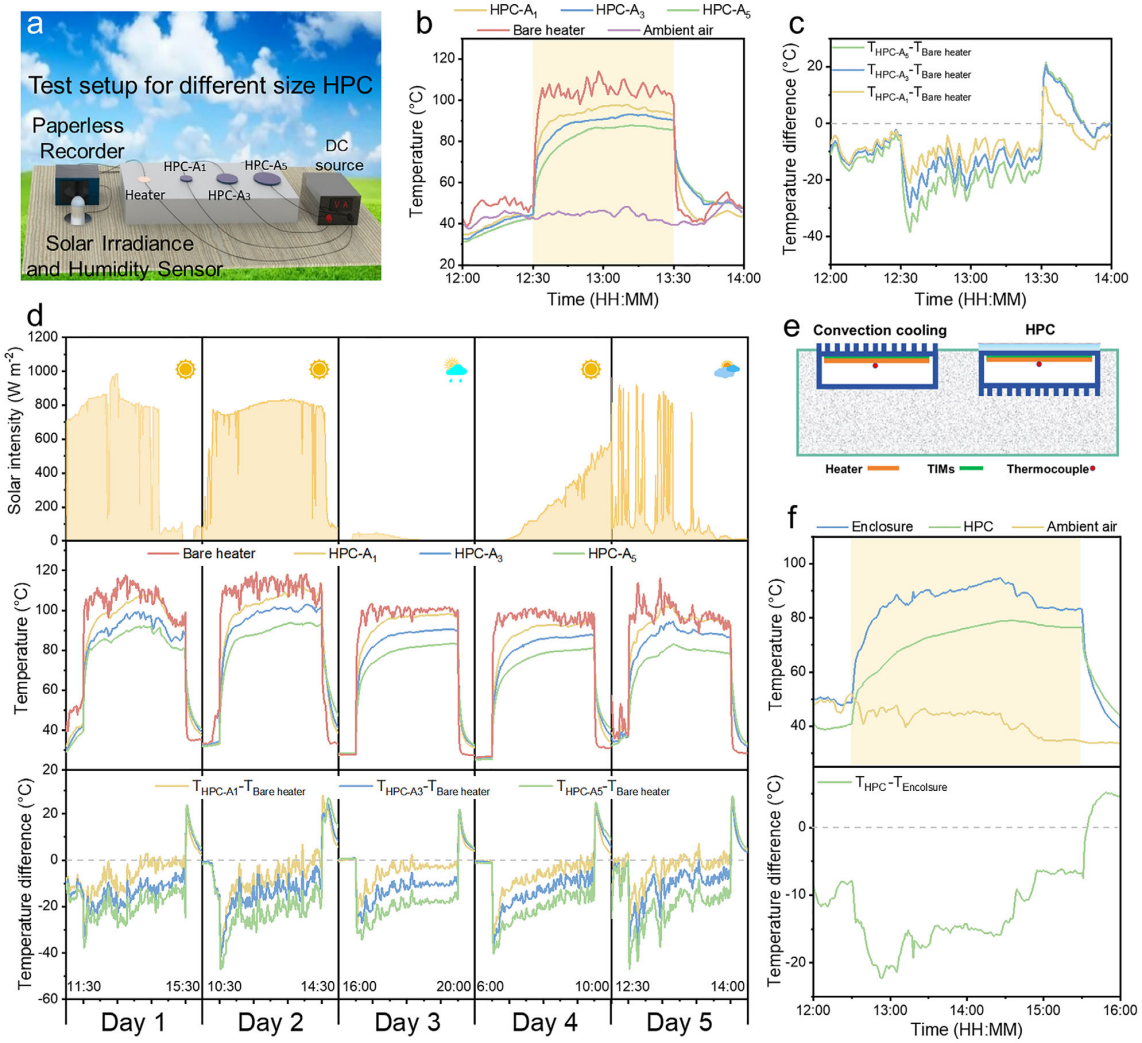
Thermal shock performance of different‐area HPC. (a) Schematic diagram of outdoor test setup for different‐area HPC. Temperature variations (b) and temperature difference (c) of different‐area HPC under 2000 W m^−2^ thermal shock with one‐hour duration. (d) A five‐day outdoor test involved daily 3‐hour thermal shocks (2000 W m^−2^) with solar radiation and temperature variations. (e) Schematic diagram of the setup for simulating the outdoor electronic with enclosures and heat source. The left side shows the fin‐based cooling mode, while the right side depicts the HPC‐integrated cooling mode. (f) Temperature variations and temperature difference of different cooling strategies under 2000 W m^−2^ thermal‐shock and solar radiation with three hours duration.

To mitigate the performance degradation of the HPC under prolonged thermal shock, we increased its area relative to the heater to enhance thermal‐shock resistance effect. Accordingly, three HPC samples with area ratios (HPC: heater) of 1:1, 3:1, and 5:1 were designed and evaluated using a self‐built test setup (Figure 5a and Figure S31). Their temperature responses to a 2000 W·m^−^^2^ thermal shock over one hour were first measured (Figure 5b,c and Figure S32). The results show a significant improvement in thermal‐shock resistance with larger area, attributed to the enhanced SH‐absorption and LH‐evaporation provided by the greater material volume. Notably, this area scaling introduces a spatial temperature uniformity consideration: for the 1:1 area ratio, heat transfers directly into the HPC without significant lateral conduction, enabling rapid localized cooling; for the 3:1 and 5:1 area ratios, heat first transfers vertically into the region directly above the heater, then spreads laterally toward outer regions (Figure S27). This lateral spreading depends on the in‐plane thermal conductivity of the HPC, which limits the heat transfer efficiency slightly but is sufficient because the primary cooling mechanisms are volumetric processes. The cooling performance does not scale strictly linearly with HPC area due to lateral heat spreading effects, but the substantially enhanced total cooling capacity from increased hydrogel volume and free water reserves ensures a net positive and significant improvement in thermal‐shock resistance. To probe the performance limits under extended exposure, the thermal shock duration was increased to three hours. Indoor tests without solar radiation (Figure S33) revealed that the cooling effect of the smallest HPC‐A_1_ nearly diminished after prolonged heating time. In contrast, HPC‐A_3_ and HPC‐A_5_ maintained effective significant cooling due to their larger free water reserves and heat capacity, with HPC‐A_5_ demonstrating optimal performance. The substantially larger cooling capacity from increased area more than compensates for the slightly reduced local heat flux concentration, as the extended area provides distributed evaporative cooling sites. COMSOL simulations (Figures S34 and S35) further confirmed that a larger HPC area provide long‐duration thermal‐shock resistance. Subsequently, a continuous five‐day outdoor test was conducted with 3 h of daily thermal shock (Figure S36). Figure 5d shows the solar irradiance, temperature, and corresponding temperature differences.

Under intense solar radiation and thermal shock, the HPC‐integrated heater experienced rapid temperature rises, HPC‐A_1_ eventually lost its cooling capacity once its free water reservoir evaporated, leading to a temperature that matched or surpassed that of the bare heater. At this stage, the free water within the PAm hydrogel was fully desorbed, and the hydrogel remained at a relatively elevated temperature. Nevertheless, the HPC maintained structural integrity under this thermal load, and upon cessation of thermal shock, it could regenerate its cooling capacity via spontaneous atmospheric moisture harvesting. In contrast, the temperatures of the heater combined with HPC‐A_3_ and HPC‐A_5_ were significantly lower than that of the bare heater, with larger areas yielding more pronounced thermal‐shock resistance. HPC‐A_5_ exhibited no significant temperature escalation or performance degradation during the sustained 3‐hour test, as the larger hydrogel volume retained sufficient free water reserves to provide continuous latent‐heat evaporative cooling. Additionally, a strong correlation was observed between the heater temperature and solar irradiance—under clear‐sky high‐irradiance conditions, all heaters exhibited elevated temperatures. This implies that in real outdoor environments, the combined effect of continuous solar radiation and intermittent thermal shocks can lead to a more pronounced temperature rise within electronic devices. The five‐day outdoor test demonstrated that the HPC not only exhibits excellent moisture harvesting capability, enabling consistent thermal‐shock resistance over multiple cycles, but also that rational area scaling can effectively mitigate thermal management challenges in outdoor electronics subjected to prolonged high‐power thermal shock. It is important to note that fluctuating wind speed and humidity dynamically influenced the cooling balance [53, 54]. Higher daytime wind speeds enhanced hydrogel evaporative cooling by reducing boundary layer resistance, while radiative cooling remained stable. Conversely, lower nighttime wind speeds favored moisture sorption for water replenishment, ensuring the HPC's adaptive thermal management under varying outdoor conditions. Finally, to evaluate practical applicability, a simulated outdoor electronic setup was constructed (Figure 5e and Figure S37) to benchmark the thermal management of a conventional finned enclosure against an HPC‐integrated one. The thermal‐shock resistance performance was evaluated by monitoring the temperature of the heater. As shown in Figure 5f and Figure S38, during the initial thermal shock, the conventional finned sink failed to provide rapid cooling via convection, while the HPC‐integrated enclosure promptly suppressed temperature rise by leveraging SH‐absorption and LH‐evaporative cooling. Moreover, the conventional enclosure, due to its weak reflectance and emittance, tended to cause excessive internal heating. Under a 3‐hour thermal shock at 2000 W·m^−^^2^, the HPC‐integrated achieved a maximum temperature reduction of 22.3°C and an average reduction of 13.8°C compared to the traditional finned convection cooling, unequivocally demonstrating its superior thermal‐shock resistance. By maintaining lower operating temperatures and mitigating dangerous thermal shocks, the HPC effectively reduces thermally induced degradation mechanisms—such as solder fatigue, dielectric breakdown, and performance throttling—thereby enhancing the operational stability and extending the service lifetime of outdoor electronic systems under realistic operating conditions. Finally, the HPC achieves excellent thermal‐shock resistance through the synergistic combination of radiative cooling, sensible‐heat absorption, and latent‐heat evaporation for outdoor electronics.

### 2.5 Ambient Cooling Performance

In the absence of thermal shock, solar radiation remains the key thermal challenge for outdoor electronics. The traditional radiative cooling materials achieve sub‐ambient cooling by reflecting sunlight and emitting mid‐infrared radiation, our proposed HPC leverages the synergistic effect of radiative cooling and LH‐evaporation for enhanced performance. Therefore, we conducted outdoor comparative tests under solar radiation using a bare heater, a TPC‐integrated heater, and an HPC‐integrated heater.

**FIGURE 6:**
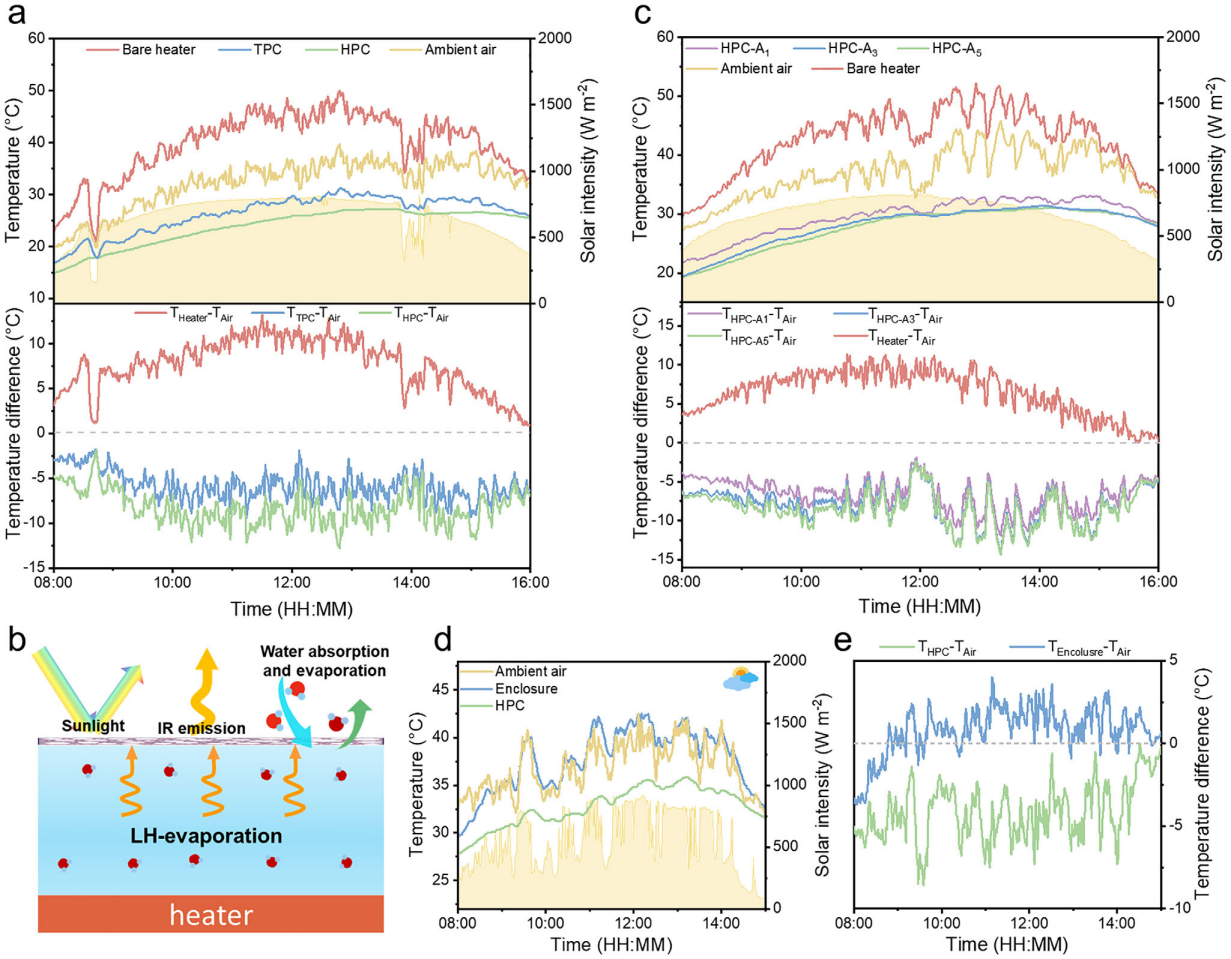
Ambient cooling performance of HPC. (a) Temperature and temperature difference of the different cooling strategies during outdoor measurement on November 22th, 2025, in Shenzhen, China. The yellow area shows the solar irradiation during the day. (b) Schematic diagram of the heat transfer model for HPC under the heater without thermal shock. (c) Temperature variation curve of different‐area HPC during outdoor measurement in November 16th, 2025. Temperature variations (d) and temperature difference (e) of different cooling strategies during outdoor measurement on October 10th, 2025.

The same self‐built apparatus (Figure 4a and Figure S21) was employed to compare the temperature variations of the heater under three cooling modes in the condition of solar radiation and no thermal shock. The temperature variations recorded on November 22th, 2025 (08:00–16:00, average solar irradiance 660.4 W·m^−^^2^) is presented in Figure 6a. A clear divergence is observed: the bare heater, devoid of solar reflection, exhibited a rapid temperature surge, stabilizing at an average of 7.7°C above ambient. Conversely, the heater integrated TPC successfully maintained a sub‐ambient state, with an average temperature drop of 5.8°C. The heater integrated HPC excelled further, maintain an average temperature of 8.3°C below ambient air. This enhanced performance underscores the exceptional capability of the HPC in delivering efficient sub‐ambient cooling for outdoor electronics exposed to solar radiation. Moreover, the HPC's ability to provide stable and superior cooling was consistently affirmed in subsequent prolonged outdoor experiments (Figures S39–S42). In the absence of thermal shock conditions, the HPC provides cooling effect to the heater through the synergistic interplay of radiative cooling and LH‐evaporation, as illustrated in Figure 6b. The porous PVDF‐HFP top layer reflects sunlight and achieves mid‐infrared emission, effectively attenuating solar heating. Meanwhile, the internal PAm hydrogel contributes additional cooling power via the LH‐evaporation. Furthermore, COMSOL simulations (Figure S43) confirm that the HPC outperforms the TPC in providing superior cooling for outdoor electronics under typical solar irradiation. In summary, the collaboration between radiative cooling and latent heat evaporation enables the HPC to achieve enhanced sub‐ambient cooling performance.

The effect of HPC area on sub‐ambient cooling performance was evaluated by comparing heaters integrated with different‐sized HPCs under solar exposure. The experimental setup shown in Figure 5a was used for a continuous two‐day outdoor test (Figures S44 and S45). Figure 6c illustrates the temperature variations on a typical sunny day (November 16th, 2025). During the period from 08:00 to 16:00 (average solar irradiance: 669.4 W·m^−^^2^), the average temperature of the bare heater exceeded the ambient temperature by 5.1°C. In contrast, heaters integrated with HPC‐A_1_, HPC‐A_3_, and HPC‐A_5_ exhibited average temperature reductions of 8.2°C, 9.6°C, and 10.0°C below ambient air, respectively. This graded improvement clearly demonstrates that increasing the HPC area effectively enhances its cooling capacity under solar radiation. Notably, the temperature rise rate of the heater integrated with HPC‐A_5_ was slightly slower than that of the heater integrated with HPC‐A_3_. This can be primarily attributed to the larger area providing greater LH‐evaporation power, which enables more robust and sustained cooling for the heater under identical solar irradiation conditions. In addition, to evaluate the thermal management capability of HPC in practical outdoor electronic devices, we compared the temperature variations of a traditional finned enclosure versus an HPC‐integrated solution using a simulated setup (Figure 5e and Figure S37) under outdoor sunlight. The results (Figure 6d,e) clearly show that the temperature of the traditional enclosure fluctuated significantly with solar exposure and was consistently higher than that of the HPC solution. During the fluctuating solar period from 08:00 to 15:00 (average irradiance: 543.4 W·m^−^^2^), the HPC‐integrated enclosure achieved an average temperature reduction of 4.1°C compared to the traditional finned enclosure. Under intense sunlight, the fins on the traditional enclosure failed to dissipate heat rapidly and effectively, resulting in its overall temperature remaining consistently above the ambient air. In contrast, the HPC, leveraging the synergistic effect of radiative cooling and LH‐evaporation, demonstrated stable and highly efficient sub‐ambient cooling performance. Therefore, the HPC provides a compelling thermal management solution for outdoor electronics under solar radiation through the synergistic combination of radiative cooling and LH‐evaporation.

## 3 Conclusion

In summary, this work addresses the critical dual thermal challenges—solar radiation and high‐power operational shocks—for outdoor electronics by proposing a self‐sustaining hybrid passive cooler (HPC). The HPC is composed of a vaporpermeable P(VdFHFP) radiative coating and a polyacrylamide (PAm) hydrogel capable of autonomous atmospheric moisture harvesting in a melamine sponge (MS) skeleton. This monolithic design creates a synergistic system that unites radiative cooling with moisture sorption–desorption thermal management capacity. Experimentally, the HPC delivers efficient subambient cooling, maintaining an averaging temperature 8.3°C below ambient air under continuous solar radiation via radiative cooling and latent‐heat evaporation. More critically, when subject to an intense 2000 W·m^−2^ thermal shock, it achieves a maximum temperature drop of 42.4°C through the combined of radiative cooling, sensibleheat absorption, and latent heat evaporation. Systematic outdoor cycling tests and simulations confirm that the HPC offers scalable cooling capacity across geometries and demonstrates reliable and stable performance over prolonged 3‐hour thermal shock, enabled by its autonomous atmospheric moisture‐harvesting capability. These advances validate the HPC as an innovative, sustainable solution for outdoor electronics thermal management.

## Author Contributions

**Qingyuan Du** have made substantial contributions to conception design of the experiments, acquisition of data, analysis and interpretation of data, and writing of the manuscript. **Meng Yang** has carried out supervisory work in experimental design, data analysis and manuscript revision. **Maoning Li** and **Yunpeng Hu** have critically revised the paper for important intellectual content. **Guangzhe Chen** and **Dandan Li** have provided critical feedback and helped shape the research, analysis, and manuscript. **Dazhi Sun** agreed to be accountable for all aspects of the work in ensuring that questions related to the accuracy or integrity of any part of the work are appropriately investigated and resolved.

## Conflicts of Interest

The authors declare no conflicts of interest.

## Supporting information




**Supporting File**: advs77183‐sup‐0001‐SuppMat.docx.

## Data Availability

The data that support the findings of this study are available from the corresponding author upon reasonable request.
