# Supplementary material for: Self‐Sustaining Hybrid Passive Cooler Enabling Highly Effective Thermal Management for Outdoor Electronics
Source: Adv Sci (Weinh). 2026 Aug 14:e77183. Online ahead of print. doi: 10.1002/advs.77183 (PMC13474183; doi:10.1002/advs.77183)
Supplement: Supplementary file 1 [file ADVS-9999-e77183-s001.docx]

***Supporting Information***

**Self-sustaining hybrid passive cooler enabling highly effective thermal management for outdoor electronics**

*Qingyuan Du, Meng Yang, Maoning Li, Guangzhe Chen, Yunpeng Hu, Dandan Li, Dazhi Sun**

Guangdong Provincial Key Laboratory of Functional Oxide Materials and Devices, Department of Materials Science and Engineering, Southern University of Science and Technology, Shenzhen, Guangdong, 518055, China

Corresponding author. Email: [sundz@sustech.edu.cn](mailto:sundz@sustech.edu.cn);

# Section 1. Materials and Methods

1. **Materials**

The melamine sponge (MS) (5 mm thickness) was obtained from Shanghai Zhenmo New Materials Ltd. The poly(vinylidene fluoride-co-hexafluoropropylene) (P(VdF-HFP)) (Kynar Flex 2801) was purchased from Arkema, French. Acetone and dimethylformamide (DMF) were obtained from Shanghai Lingfeng Chemical Reagent Co., Ltd. The acrylamide (Am) was purchased from Shanghai Aladdin Biochemical Technology Co., Ltd. N,N'-Methylenebisacrylamide (MBAA) was purchased from Shanghai Aladdin Biochemical Technology Co., Ltd. Ammonium persulfate (APS) was purchased from Tianjin Damou Chemical Reagent Factory. N,N,N',N'-tetramethylethylenediamine (TEMED) purchased from Shanghai Aladdin Company. Polyethylene glycol (PEG, Molecular weight 2000) was obtained from Shanghai Aladdin Company.

2. **Preparation of** **PVDF-HFP@MS and PVDF-HFP/PEG@MS Skeleton-coating**

Before spraying on the surface of MS, MS was soaked in anhydrous ethanol solution, then ultrasonically treated for 10 minutes with an ultrasonic instrument (Kunshan Hechuang Qusheng Instrument Co., Ltd., model KH3200DE), and then dried in oven. The detail preparation process of P(VdF-HFP)@MS and P(VdF-HFP)/PEG@MS is shown in **Figure S7**.

Preparation of P(VdF-HFP)@MS skeleton-coating: P(VdF-HFP) powder was first dissolved in Acetone/DMF (1:1) solution, and then had water added to make the P(VdF-HFP)-Acetone-water precursor solution with a 1:8:1 mass ratio. Subsequently, the precursor solution was sprayed onto the surface of MS. Finally, after the Acetone/DMF and water have evaporated, the P(VdF-HFP)@MS skeleton-coating integrated material was obtained.

Preparation of P(VdF-HFP)/PEG@MS skeleton-coating: The P(VdF-HFP) was first dissolved in an Acetone/DMF (1:1) solution, and a water-PEG solution with a ratio of 1:0.2 was prepared. Then, the water-PEG solution was added to make a P(VdF-HFP)-Acetone/DMF-water-PEG precursor solution with a 1:8:1:0.2 mass ratio. The precursor solution was then sprayed onto the surface of MS. Next, the Acetone/DMF and water have evaporated, the material was immersed in heated water with gentle stirring to remove the internal PEG. After drying, the P(VdF-HFP)/PEG@MS skeleton-coating integrated material is obtained. By introducing soluble PEG into the water phase, more pores can be incorporated into the P(VdF-HFP) coating, thereby enhancing the scattering and water vapor permeability of the coating structure.

The thickness of the P(VdF-HFP) coating on the MS skeleton surface prepared in the above process was controlled at approximately 200 μm. This could ensure both effective mechanical interlocking between the P(VdF-HFP) coating and the MS scaffold and provides pathways for the movement of free water within the hydrogel in the subsequent.

3. **Preparation of TPC and HPC**

Preparation of traditional passive cooler (TPC)^[1]^: P(VdF-HFP) is first dissolved in an acetone/DMF (1:1) solution, then water is added to prepare a P(VdF-HFP) acetone-water precursor solution with a mass ratio of 1:8:1. The precursor solution is then sprayed onto a release film to obtain the desired film thickness. Finally, after the acetone/DMF and water have evaporated, the P(VdF-HFP) film was obtained, which was denoted as TPC.

Preparation of hybrid passive cooler (HPC): Based on the P(VdF-HFP)/PEG@MS samples prepared in Section 1.2, the PAm hydrogel was further infiltrated the P(VdF-HFP)/PEG@MS skeleton-coating to obtain P(VdF-HFP)/PEG@MS-PAm samples, which was denoted as HPC. The preparation process was as follows: First, 3 g of Am monomer and 7 g of deionized water were weighed. Subsequently, progressively add crosslinking agent (MBAA) and initiator (APS) at concentrations of 0.5% and 1% by weight of the Am monomer to dissolve, respectively. Next, accelerator (TEMED) accounting for 0.5% of the water mass was added, and stirring was continued for 10 minutes.

Then, the P(VdF-HFP)@MS or P(VdF-HFP)/PEG@MS sample was immersed in the mixed solution, ensuring that the solution fully infiltrated the MS framework. Meanwhile, nitrogen gas was purged and PE film was used to isolate oxygen, preventing oxygen from inhibiting the free radical reaction. Finally, the entire setup was transferred to an oven at 65°C for constant temperature heating for 3 hours to complete cross-linking polymerization to obtain P(VdF-HFP)@MS-PAm and P(VdF-HFP)//PEG@MS-PAm samples, respectively. The samples were then immersed in deionized water for 24 hours (with water changed 3 times during this period) to remove residual reagents.

The above samples are composed of an MS skeleton base with a tandem structure of a P(VdF-HFP) radiative cooling coating and a PAm hydrogel evaporation-latent heat storage integrated structure. The P(VdF-HFP)@MS-PAm and P(VdF-HFP)//PEG@MS-PAm samples are named normal HPC (N-HPC) and porous HPC (P-HPC), respectively.

4. **Characterizations**

The morphology of the h-BN/PDMS composite was characterized by using a field-emission scanning electron microscope (FE-SEM, Tescan Mira 3) operating with a 5 kV accelerating voltage. The internal pore size distribution of the two samples, PVDF-HFP@MS and PVDF-HFP/PEG@MS, was tested and analyzed using a capillary pore size analyzer (Porolux 100, Germany). The water vapor transmission rate of different samples was tested using a water vapor transmission rate tester (China-Luozhong-RL450, GB/T 12704.1-2009). The surface wettability was measured by a contact angle meter (VCA optima, USA) via static water contact angle (CA) and sliding angle (SA) measurements, which were carried out by using 8 μL water droplet and over three independent tests.

The spectral reflectivity (in the sunlight region, 0.3-2.5 μm) of cooler samples was measured by a UV–vis–NIR spectrophotometer (PerkinElmer Lambda 950s) with a polytetrafluoroethylene integrating sphere. The average solar reflectance was determined by weighted calculation based on the AM1.5 solar spectrum, as follow:

$$\bar{R}_{solar}=\frac{\int_{0.3um}^{2.5um} I_{AM1.5}(\lambda)R(\lambda)d\lambda}{\int_{0.3um}^{2.5um} I_{AM1.5}(\lambda)d\lambda}$$

The spectral emissivity (in the mid-infrared wavelength range, 8-13 μm) of cooler samples was measured by Fourier transform infrared spectrometer (FTIR, Bruker Vertex 70v) equipped with a gold integrating sphere. The average emissivity located at atmospheric window was determined by weighted calculation based on the blackbody radiation function, as follow:

$$\bar{\varepsilon}_{IR}=\frac{\int_{8um}^{13um} I_{BB}(\lambda,T)\varepsilon(\lambda)d\lambda}{\int_{8um}^{13um} I_{BB}(\lambda,T)d\lambda}$$

where 𝜆 is the wavelength, is the *I_AM1.5_*(𝜆) is the AM 1.5 global solar intensity, and 𝑅(𝜆) is the surface solar reflectivity at 𝜆. Addition, *I_BB_*(𝜆, *T*) is the spectral radiance of a blackbody at temperature T and wavelength 𝜆, h is Planck’s constant, c the 𝜆 speed of light in vacuum and is the Boltzmann constant. *I_BB_*(𝜆, *T*) was calculated as follow:

$I_{BB}\left( \lambda,T \right)=\frac{2hc^{2}}{\lambda^{5}}*\frac{1}{e^{\frac{hc}{\lambda k_{B}T}}-1}$

*𝜀*(𝜆) is the surface thermal emissivity at wavelength (𝜆), which was calculated as follow:

$$\varepsilon\left( \lambda\right)=A\left( \lambda\right)=1-R\left( \lambda\right)-T\left( \lambda\right)$$

where is the *A*(𝜆)absorptivity and *T*(𝜆) is the transmittance.

The thermal stability was measured by the Thermogravimetric analyzer (TGA, METTLER TA55, Switzerland), at a temperature range from 25 °C to 800 °C and a scanning rate of 10 °C min^-1^ under N_2_ atmosphere. The absorbing heat process of the different HPC samples investigated by the differential scanning calorimeter (DSC, METTLER 1, Switzerland) under the N_2_ atmosphere with temperature range from 20-200 °C and temperature change rate of 10 °C min^-1^. Sapphire testing (Netzsch STA449F3, Germany) was employed to determine the specific heat capacity changes of HPC-free samples between 20-100°C.

The tensile strength of the HPC was measured by a universal instrument (Zhiqu ZQ-990-2, China). The sample was 5 cm long and 1 cm wide with a gauge distance of 3 cm, and the test speed was 5 mm min^-1^. Each sample was tested three times and take the average value.

To evaluate the moisture absorption performance of the HPC, the HPC samples were immersed in LiCl solutions of varying concentrations (0, 15, 30, and 45 wt%), and named HPC_0_, HPC_15_, HPC3_0_, and HPC_45_. After complete swelling, LiCl was embedded into the cross-linked network of PAm hydrogel. The samples were weighed and then dried at 80 °C for 6 hours to remove free water (**Figure S12**). The residual weight represents the dry weight of the sample, as well as the weight of LiCl and bound water. The process of moisture loss upon heating and moisture absorption at room temperature for the samples was tested using a hot stage for heating and a constant temperature and humidity chamber (HSX-450, Zhongyi Guoke (Beijing) Technology Co., Ltd.) for moisture absorption. The samples were heated for 60 minutes, and the mass changes were recorded at different heating times. The samples were then transferred to the chamber at 25°C and 60% RH for 150 minutes of moisture absorption. The sorption kinetics of the samples under different relative humidities were measured using the constant temperature and humidity chamber, with temperature and relative humidity accuracies of ±0.5 °C and ±3% RH, respectively. Prior to sorption testing, all samples were dried at 80 °C for 6 hours to remove free water. Once the test chamber reached the set temperature (25 °C) and humidity levels (30%, 60%, and 90% RH), the dried samples with an area of 10 mm² were transferred into the chamber for moisture absorption measurements. Mass changes were recorded by an electronic balance (METTLER TOLEDO ME104). The mass change process during absorption was measured using the same method for the samples with different areas. The moisture absorption and heating weight loss test procedure involves placing the sample in a constant temperature and humidity chamber at 90% relative humidity for 24 hours to absorb moisture. The sample is then transferred to an oven at different temperatures for 3 hours of baking, with mass changes recorded at various heating durations. The sample underwent 40 cycles of moisture absorption testing using the same test method.

5. **Moisture Adsorption and Desorption Tests of Bulk Sample**

5.1 Moisture adsorption

The moisture adsorption was monitored and evaluated using a precision electronic balance in a controlled environment at 25°C with relative humidity ranging from 30% to 90% (Sample sizes: 10×10×5 mm, 24-hour test period with recording intervals of 1 or 2 hours). The HPC with different LiCl loadings exhibited distinct moisture adsorption characteristics. Since HPC is designed to adhere to electronic device surfaces in practical applications, where the bottom interface presents significant mass transfer resistance to water vapor, the samples were tested on glass substrates to accurately simulate real-world operating conditions and reflect their actual adsorption performance. The water uptake content ($W_{t}$) was calculated using the following equation:

$W_{t}=\frac{m_{t}-m_{\text{dry}}}{m_{\text{dry}}}$	(S1)

Where $m_{t}$ and $m_{\mathrm{dry}}$ are the mass of HPC after and before water vapor adsorption.

To assess the potential influence of surface-area-to-volume ratio—which varies with sample size—on moisture absorption, a systematic evaluation of HPC_45_ sample with different surface areas was conducted. Three sample sets were designed with planar dimensions of 10 mm × 10 mm, 50 mm × 50 mm, and 100 mm × 100 mm, each having a thickness of 5 mm. The water absorption rate was computed according to Equation S1 mentioned previously.

5.2 Moisture desorption

The moisture desorption of the HPC was measured using a constant-temperature hot plate. This parameter is influenced by both the material temperature and its current water content, resulting in a complex relationship. In the first set of experiments, HPC samples with different LiCl loadings were heated at 80 °C for 1 h and then transferred to a climate chamber at 25 °C and 60% RH for moisture adsorption. Mass changes were recorded throughout the heating-evaporation-adsorption process. In a separate experiment, HPC_45_ samples were first saturated in a 90% RH environment and then placed on hot plates at 60 °C, 80 °C, and 100 °C, respectively, to determine the water evaporation rates during desorption under different temperatures.

To further evaluate and theoretically validate the thermal management performance of the proposed HPC, water evaporation rate tests were conducted on HPC samples without LiCl addition. To simulate the desorption behavior of moisture within the HPC under realistic thermal shock conditions in outdoor electronics, multiple temperature points in the range of 25–100 °C were selected (under laboratory conditions with 60±3% RH and 25±0.5 °C, without considering additional factors such as wind speed or atmospheric pressure). At each temperature, the samples were subjected to 30 minutes of constant heating, and the corresponding evaporation rates were recorded. All samples had uniform dimensions of 100×100×5 mm, and each temperature condition was tested in triplicate with results averaged. The fitting results demonstrate excellent agreement between the measured evaporation rates at individual temperature points and the fitted curve, with a correlation coefficient approaching 99%. The obtained data were used to fit the water evaporation parameters of the HPC, thereby establishing the functional relationship between evaporation rate and temperature for subsequent cooling power calculations.

The fitting process and results are as follows:

**Table SⅠ**. Evaporation rate results under different hotplate temperatures

| Temperature (°C) | Evaporation rate (g/min·cm^-2^) |
| --- | --- |
| 25 | 0.000122±0.000036 |
| 30 | 0.000246±0.000054 |
| 40 | 0.000516±0.000048 |
| 50 | 0.00091±0.000124 |
| 60 | 0.00164±0.00024 |
| 70 | 0.002433±0.00027 |
| 80 | 0.003821±0.00031 |
| 90 | 0.005452±0.00025 |
| 100 | 0.006857±0.00023 |

Based on the above data, parameter fitting was performed using MATLAB software. The relationship between temperature and evaporation rate was fitted using a power function, with the fitting process as follows:

*% ===============================*

*% Power function fitting of hydrogel evaporation rate vs temperature*

*% R(T) = a * T^b*

*% ===============================*

*% Original data*

*T = [25 30 40 50 60 70 80 90 100]; % Temperature (°C)*

*R = [0.000122 0.000246 0.000516 0.00091 0.00164 0.002433 0.003821 0.005452 0.006857]; % Evaporation rate*

*% ---------- Power function fitting ----------*

*% Log transformation: ln(R) = ln(a) + b*ln(T)*

*logT = log(T);*

*logR = log(R);*

*% Linear fitting for parameters*

*p = polyfit(logT, logR, 1);*

*b = p(1);*

*a = exp(p(2));*

*% Display fitting parameters*

*fprintf('Power law fitting result: R(T) = %.3e * T^{%.3f}\n', a, b);*

*The results of the power law function fitted to the raw data are as follows:*

$$R\left( T \right)=0.0000000126\times T^{2.875}$$

*Where:*

*a ≈ 0.0000000126*

*b ≈ 2.875*

**Figure S****Ⅰ.** Evaporation rates and fitted curves of HPC at different temperatures.

6. **Cooling Power Calculation of HPC**

Outdoor electronics primarily operate under two typical thermal scenarios: one involves heating solely from solar radiation without internal thermal shock, while the other occurs during high-power operation, where heat generated by internal chips induces thermal shock, causing a rapid temperature rise that propagates to the HPC, leading it to undergo heating until reaching thermal equilibrium. Therefore, analyzing the cooling power of the HPC requires clear differentiation between these two operational states.

As illustrated in **Figure SⅡa**, when outdoor electronics are not subjected to thermal shock, the HPC primarily provides cooling power through radiative cooling and low-temperature LH-evaporation, maintaining the device at sub-ambient temperature levels. The LH-evaporation power is predominantly governed by the evaporation rate, which is determined by ambient temperature. While, when the outdoor electronics experience thermal shock, the HPC consequently heats up and eventually reaches a higher equilibrium temperature, with its dynamic temperature rise process depicted in **Figure SⅡb**. The cooling mechanism of the HPC during this heating process requires further detailed analysis: in the initial stage, as the temperature increases, the hydrogel material contributes to cooling through both SH-absorption and LH-evaporation. Once thermal equilibrium is attained, the SH-absorption effect ceases, and the system continues to dissipate heat solely via LH-evaporation at elevated temperatures. Thus, the total cooling power provided by the HPC during thermal shock process reaches its maximum at the temperature equilibrium point. This peak power consists of three components: radiative cooling power, SH-absorption power, and LH-evaporation power, which together serve as a theoretical basis for evaluating the thermal management capability of outdoor electronics under thermal shock conditions.

Based on the above analysis, this study will theoretically calculate and evaluate the cooling power of the HPC under two typical operating conditions: one under no thermal-shock conditions, where radiative cooling and low-temperature LH-evaporation are considered; the other during thermal shock events, where the combined contributions of radiative cooling, SH-absorption, and high-temperature LH-evaporation are integrated, using the temperature equilibrium point as the theoretical reference for calculating the maximum cooling power.

**Figure S****Ⅱ.** Schematic of the HPC temperature curve during no thermal shock (a) and thermal shock (b) processes.

6.1 Radiative cooling power calculation

The HPC provides for radiative cooling power calculations using the same fundamental formula as for other materials:

$P_{\text{radiative cooling}}=P_{\text{rad}}-P_{\text{a}\text{tm}}-P_{\text{Sun}}-P_{con}$	(S2)

where *P_rad_* indicates the thermal radiation power provided by the cooler, which can be expressed as follow:

$P_{rad}=\sigma\varepsilon_{HPC}{T_{HPC}}^{4}$	(S3)

*P_amb_* refer to the absorption of ambient air radiation power, which can be determined as follow:

$P_{atm}=\sigma\varepsilon_{air}\varepsilon_{HPC}{T_{amb}}^{4}$	(S4)

*P_sun_* is the absorbed solar irradiation power by the sample, which can be expressed as follow:

$P_{sun}=(1-R_{HPC})\times I_{AM1.5}(\lambda)$	(S5)

*P_non-radiative_* is the non-radiative heat power, which including heat conduction and convection. It can be calculated as follow:

$P_{con}=h_{non-radiative}(T_{amb}-T_{HPC})$	(S6)

where *h_non-radiative_* is the non-radiative heat transfer coefficient. In this work, we set the value as 10 W m^-2^ K^-1^ for theoretical calculation.

6.2 No thermal shock conditions cooling power calculation

Under air ambient temperature (set temperature range: 25–45 °C) and humidity conditions, the MS-PAm hydrogel within the HPC continuously absorbs and dissipates heat through water evaporation and radiative cooling through top layer, maintaining its temperature below the ambient wet-bulb level. In this steady state, the cooling performance of the HPC is primarily contributed by radiative cooling and low-temperature LH-evaporation. The total net cooling power can be expressed as:

$P_{total}=P_{radiative cooling}+P_{LH-Evaporation}$	(S7)

$P_{LH-Evaporation}$ is the cooling power provided by the LH-evaporation of the HPC during air ambient temperature^[2]^, calculated as follows:

$P_{LH-Evaporation}=\frac{Q_{LH-Evaporation}}{t}$	(S8)

where $Q_{LH-Evaporation}$ is the LH-evaporation energy of the HPC, $m_{water evaporated}$ is the mass of water evaporated by the HPC, $\Delta H_{vap}\left( T \right)$ is the enthalpy of vaporization of HPC at temperature *T*.

$Q_{LH-Evaporation}=m_{water evaporated}\times\Delta H_{vap}(T)$	(S9)

The evaporated water mass of the HPC is related to the evaporation area and time, and the evaporation rate is a function of temperature. Therefore, the evaporated water mass of the HPC is obtained through the following calculation:

$m_{water evaporated}= Evaporation Rate(T)\times A\times t$	(S10)

where $Evaporation Rate(T)$ is the evaporated mass per unit area per time (obtained by fitting Figure S1 above), *A* represents the evaporation area of the HPC, and *t* denotes the evaporation time. The evaporation rate formula is as follows:

$Evaporation Rate\left( T \right)=0.0000000126\times T^{2.875}$	(S11)

The enthalpy of evaporation of water in HPC was calculated using empirical equations:

$\Delta H_{vap}\left( T \right)=(2501-2.36\times T)$	(S12)

The theoretical analysis above clearly demonstrates the cooling contributions of the HPC through radiative cooling and evaporative latent heat for outdoor electronics under no thermal-shock conditions.

6.3 Thermal shock conditions cooling power calculation

Under sustained thermal shock conditions in outdoor electronics, the device temperature rises rapidly, causing the temperature of the attached HPC to also increase through thermal conduction. The corresponding temperature rise curve is shown in Figure S2 (set temperature range: 25–100 °C). During this thermal shock process, the cooling power provided by the HPC comprises three components: SH-absorption by the HPC during the heating process, continuous LH-evaporation, and radiative cooling power. Thus, the total net cooling power in this stage can be expressed as:

$P_{total}=P_{radiative cooling}+P_{SH-Absoraption}+P_{LH-Evaporation}$	(S13)

where $P_{SH-Absoraption}$ is the power of SH-absorption of HPC during the heating process^[3]^. The cooling power calculations for radiative cooling and LH-evaporation are shown previously.

To quantify the SH-absorption of the HPC during thermal shock process, this study focuses on the representative phase where the system heats up to the maximum equilibrium temperature within 30 minutes. The average SH-absorption cooling power of the HPC during this period was calculated, with results presented below:

$P_{SH-Absoraption}=\frac{mC_{\rho HPC}\left( T_{final}-T_{initial} \right)}{A*t}$	(S13)

where $m_{HPC}$is the mass per unit area of the HPC, ${C_{\rho}}_{HPC}$is the specific heat capacity of the HPC, *A* is the unit area of the HPC, and *t* is the time of SH-absorption during thermal shock process.

**Table SⅡ.** Parameters for the thermal and cooling power calculation

| **Parameters** | **Definition** | **Value** | **Unit** |
| --- | --- | --- | --- |
| ɛ_HPC_ | Emissivity of HPC | 0.96 | / |
|  | Stefan-Boltzmann constant | 5.67*10^-8^ | W m^-2^ K^-4^ |
| T_cooler_ | Temperature of the cooler | Calculated | / |
| ɛ_air_ | Emissivity of air | 0.724[4] | / |
| T_air_ | Ambient air temperature | 298 | K |
| I_AM1.5_ | Standard solar radiation | 1000 | W m^-2^ |
| R_HPC_ | Reflectance of cooler | 0.94 | / |
| $m_{water evaporated}$ | Evaporated Water Mass in HPC | / | g |
| $\Delta H_{vap}(T)$ | Evaporation Rate of HPC at Different Temperatures | Calculated | g/min·cm^-2^ |
| $A$ | The HPC area of the experiment | 100*100 | mm |
| $T$ | The temperature of HPC | / | ℃ |
| $C_{\rho_{HPC}}$ | Specific heat capacity of  the HPC | 3.87 | J/g ℃ |
| $m_{HPC}$ | The mass of HPC in testing | 51.875 | g |

**7. Radiative Cooling and Thermal-shock Resistance Properties**

The outdoor environmental evaluation of the HPC was conducted in Shenzhen, China (22°32'48"N, 114°03'16"E) from July to December 2025. To accommodate different experimental requirements, multiple uncovered test setups with varying dimensions were designed (shown in Figures 4a, 5a, and 6, respectively). Each heater was attached to a polystyrene foam base wrapped with aluminum foil to effectively isolate the setup from ambient thermal disturbances. It should be noted that all samples were directly exposed to ambient air on their top surfaces to enable sufficient moisture exchange between the HPC and the environment, thereby closely simulating the thermal conditions of outdoor electronic devices.

In the experiment shown in Figure 4a, the simulated outdoor electronics used a heater with a diameter of 70 mm, and both the conventional radiative cooler and the HPC were sized to match this dimension. For the study in Figure 5a, which aimed to evaluate the thermal-shock resistance and cooling performance of HPCs with different surface areas, the heater diameter was reduced to 30 mm. Corresponding to area ratios of 1:3:5 relative to the heater, three HPC samples were prepared with diameters of 30 mm, 52 mm, and 67 mm, respectively.

To further validate the thermal management performance of the proposed HPC in outdoor electronics, we constructed a comparative experimental setup as shown in Figure 5e, based on the typical structure of outdoor electronic products. The mock-up device measures 150 mm×80 mm×50 mm and houses a 70 mm diameter heater secured with thermally conductive adhesive tape to simulate the operational state of a chip, with its temperature monitored in real time by thermocouples. The left side of the setup employs commonly used cooling fins for passive air convection heat dissipation, while the right side incorporates the HPC attached to the heat dissipation surface for thermal management. By comparing the temperature variations of the fin-based and the HPC under identical thermal process, this study clearly demonstrates the advantages of the HPC, thereby paving a new pathway for the thermal management of future high-power outdoor electronics.

7.1 Thermal-shock resistance properties

In evaluating the thermal-shock resistance performance of the HPC, a DC power supply was used to energize the heaters. The target thermal-shock power was first determined according to the heater model and then achieved by adjusting the applied DC voltage. The required operating voltage for heater of different sizes and specifications can be determined using the follow Equation S14. This study employed two main types of heaters with the following basic parameters: one with a diameter of 70 mm, a rated voltage of 12 V, and a rated power of 13 W; and another with a diameter of 30 mm, a rated voltage of 24 V, and a rated power of 50 W. A temperature sensor mounted at the bottom of the heater recorded the temperature variations in real time, while other relevant parameters were collected following the procedures described in Section 7.2.

$U=U_{e}\sqrt{\frac{Q\pi d^{2}}{4P_{n}}}$	(S14)

where $U$ (unit: V) is the target voltage to be applied, $U_{e}$ (unit: V) denotes the heater's rated voltage, $Q$ (unit: W/m²) represents the target heat flux density, $d$ (unit: m) indicates the heating pad diameter (mm), and $P_{n}$ (unit: W) signifies the heating pad's rated power.

7.2 Sub-ambient cooling properties

In evaluating the cooling performance of the materials, the heater in the test setup remained switched off. The cooling effect was assessed by measuring the temperature at the bottom surface of the heater. Prior to testing the sub-ambient cooling performance, the HPC was preconditioned by exposing it to natural outdoor conditions for 12 hours to reach equilibrium temperature and humidity with the environment. This pretreatment aimed to simulate the realistic application scenario of the HPC and prevent potential moisture uptake deviations caused by artificial environmental control. Ambient temperature, humidity, and solar irradiance were monitored in real time using a temperature-humidity sensor (TH2XR, Shenzhen Huahanwei Technology Co., Ltd.). Wind speed data is obtained from the website of the Shenzhen Municipal Meteorological Bureau. Specifically, the test setup compared the sub-ambient cooling performance of three types: a bare heater, a heater covered with conventional radiative cooling material, and a heater integrated with the HPC.

8. **Simulations**

8.1 Optical simulation

We analyzed the sunlight scattering capability of PVDF-HFP film materials in three-dimensional space using the Finite-Difference Time-Domain (FDTD) method. The calculations were performed by Lumerical FDTD Solutions software. A simple two-dimensional model was adopted to facilitate calculations; as shown in Figure S10, the total field-scattered field (TFSF) source was used to simulate the scattering cross-sections of PVDF-HFP with different pore sizes. The total field-scattered field (TFSF) source and perfectly matched layer (PML) were employed as boundary conditions. The simulated scattering data were collected by a monitor.

8.2 Thermal simulation

Thermal simulations were performed using COMSOL Multiphysics 6.1 to analyze the temperature evolution of a heater under different cooling materials. The model accounted for both solar radiation and thermal shock generated by the heater. The heater was configured with a diameter of 50 mm and a thickness of 1 mm. Both the TPC and HPC shared the same planar dimensions as the heater, with the HPC and TPC thicknesses set to 5 mm and 0.3 mm, respectively. All coolers were placed above the heater. The following section details the COMSOL modeling methodology using the HPC as a representative case. It should be noted that the simulated temperature output represents the average temperature of the heater rather than that of the cooler, aligning with realistic testing conditions. Heat generated by the heater is conducted into the HPC, which is subsequently influenced by the following four thermal effects:

1. HPC receives heat flux from solar radiation while radiating heat to the environment. This process is calculated based on the multispectral emissivity values of HPC.
2. Moisture desorption from HPC during the heat absorption process. A coupled model of heat transfer and mass transfer in porous media was used to simulate this process, and the simulation calculation was performed with the support of experimentally measured data.
3. The surface and side surfaces of HPC are affected by convective heat flux, which was simulated using an external natural convection model.
4. During the thermal shock of the heater, the generated heat is directly transferred to HPC through thermal conduction.

The specific parameters are summarized in the table below:

Table SIII. Parameters set for thermal simulation via COMSOL Multiphysics 6.2

| Parameter | Value or expression | Unit | Description |
| --- | --- | --- | --- |
| $\varepsilon_{\text{heater}}$ | $0.6,\lambda\in(0.3,2.\text{5 }\text{μ}\text{m})$ $0.8,\lambda\in(2.\text{5},18 \text{μ}\text{m})$ | 1 | Emittance of the heater in different wavelength |
| $\varepsilon_{\mathrm{TPC}}$ | $0.05,\lambda\in(0.3,2.\text{5 }\text{μ}\text{m})$ $0.85,\lambda\in(2.\text{5},18 \text{μ}\text{m})$ | 1 | Emittance of the TPC in different wavelength |
| $\varepsilon_{\text{HPC}}$ | $0.05,\lambda\in(0.3,2.\text{5 }\text{μ}\text{m})$ $0.95,\lambda\in(2.\text{5},18 \text{μ}\text{m})$ | 1 | Emittance of the HPC in different wavelength |
| $\lambda_{\text{HPC}}$ | $\left( \begin{matrix} 0.5 & 0 & 0 \\ 0 & 0.5 & 0 \\ 0 & 0 & 0.5 \end{matrix} \right)\text{ }$ | W m^-1^ K^-1^ | Thermal conductivity of HPC in x, y and z directions |
| $C_{\text{p,HPC}}$ | 3500 | J kg^-1^ K^-1^ | Specific heat capacity of HPC |
| $\rho_{\text{HPC}}$ | 1000 | kg m^-3^ | Density of HPC |
| $W_{\text{HPC}}$ | 0.9 | 1 | Saturated HPC dry basis water content |
| $\Delta H_{\text{evap}}$ | 2200 | J g^-1^ | Enthalpy of evaporation of water in HPC |
| $P_{heater}$ | 2000 | W m^-2^ | Thermal-shock power of the heater during thermal shock process |

The model used for simulating different cooling modes of the heater in COMSOL is shown in Figure S, and its shape and structure are consistent with those of the heater used in the experiment. In addition, thermal radiation was adopted to represent solar irradiation during the simulation, while the thermal shock process of the heater was simulated in the form of thermal conduction. The environmental conditions used in the simulation were derived from the meteorological station data provided by COMSOL (ASHRAE 2021, Shenzhen, China). Further, the temperature variations during thermal shock for the heater combined with different areas of HPC was simulated by varying their diameter in the next simulation model.

# Section 2. Supporting tables and figures

**Figure S1**. A typical outdoor electronic of 5G base station schematic diagram.

**Figure S2.** Cooling principle of the HPC under two typical application scenarios. The unit of cooling power is watt per square meter (W m^−2^).

**Figure S3.** The cooling power contribution of the HPC under no thermal-shock conditions comprises two components: (a) latent heat evaporation cooling power, and (b) radiative cooling power.

**Figure S4.** The cooling power contribution of the HPC under thermal-shock conditions comprises two components: (a) latent heat evaporation cooling power, (b) sensible heat absorption cooling power, and (c) radiative cooling power.

**Table S1.** Specific information of Figure S5 in the following text: performances evaluation indicators

| **Performances** | **Descriptions** | **Scoring scheme** | |
| --- | --- | --- | --- |
| **Thermal-shock resistance** | The cooling effect of the cooling material on the heater under a 30-minute thermal shock of 2000 W·m⁻² | **5** | **≥**40 ℃ |
|  |  | **4** | **≥**30 ℃ |
|  |  | **3** | **≥**20 ℃ |
|  |  | **2** | **≥**10 ℃ |
|  |  | **1** | **≥**0 ℃ |
| **Sub-ambient cooling** | The stable temperature that a radiative cooling material can reach at an ambient temperature of 35 °C and a sunlight irradiation of 1000 W·m⁻² | **5** | <27 ℃ |
|  |  | **4** | <30 ℃ |
|  |  | **3** | <33 ℃ |
|  |  | **2** | <35 ℃ |
|  |  | **1** | **≥**35 ℃ |
| **Cooling power** | The cooling power provided by the material during thermal shock process | **5** | **≥**2.5 kW m^-2^ |
|  |  | **4** | **≥**2 kW m^-2^ |
|  |  | **3** | **≥**1.5 kW m^-2^ |
|  |  | **2** | **≥**1 kW m^-2^ |
|  |  | **1** | <0.5 kW m^-2^ |
| **Cyclic stability** | The cooling effect of the cooling materials on the heater after cyclic thermal shock testing | **5** | **≥**40 ℃ |
|  |  | **4** | **≥**30 ℃ |
|  |  | **3** | **≥**20 ℃ |
|  |  | **2** | **≥**10 ℃ |
|  |  | **1** | **≥**0 ℃ |
| **Weather resistance** | The material exhibits excellent hydrophobic performance, enabling environmental adaptability. | **5** | **≥**135 ° |
|  |  | **4** | **≥**120 ° |
|  |  | **3** | **≥**110 ° |
|  |  | **2** | **≥**90 ° |
|  |  | **1** | <90 ° |

**Table S2**. Specific information of Figure S5 in the main text: samples information

| **Sample name** | **Material** | **Reference** |
| --- | --- | --- |
| **Radiative cooling** | P(VdF-HFP) | ^[1]^ |
| **Radiative cooling + PCMs** | PDMS/h-BN@PW | ^[4]^ |
| **Hydrogel** | Sodium polyacrylate | ^[5]^ |
| **HPC** | P(VdF-HFP)@MS-PAm | This work |

**Table S3.** Comparation of HPC with various cooling materials

|  | Cooling material | *R*_Solar_ | *E*_MIR_ | *W*_sat_ (g g^-1^)* | Reference |
| --- | --- | --- | --- | --- | --- |
| **1** | **HPC** | **0.96** | **0.95** | **1.75** | **This work** |
| 2 | CCP | 0.93 | 0.95 | 0.3 | *Science* **2025**^[6]^ |
| 3 | PVA/BaSO_4_ | 0.87 | 0.97 | 0.5 | *Nano Lett.* **2024**^[7]^ |
| 4 | PAAS | 0.93 | 0.99 | 0.3 | *Nat. Comm.* **2023**^[5]^ |
| 5 | PVA/CA | 0.93 | 0.94 | 0.3 | *Sci. Adv.* **2022**^[8]^ |
| 6 | P(VDF-HFP) | 0.96 | 0.97 | -** | *Science* **2018**^[1]^ |
| 7 | PMP | 0.97 | 0.85 | -** | *Science* **2024**^[9]^ |
| 8 | Cooling glass | 0.96 | 0.95 | -** | *Science* **2023**^[10]^ |
| 9 | Al_2_O_3_ | 0.99 | 0.96 | -** | *Science* **2023**^[11]^ |

*: To standardize adsorption measurements across various articles and material thicknesses, we restricted the material thickness to 5 mm, comparable to the HPC.

**: No relevant data.

**Figure S5.** A comprehensive comparison of HPC and other outstanding passive cooling materials.

**Figure S6**. Schematic of the HPC preparation process.

**Figure S7.** Comparison of two P(VdF-HFP) coatings prepared by phase separation methods. (a) Schematic and SEM image of P(VdF-HFP) coating prepared by aqueous phase separation. (b) Schematic and SEM image of porous P(VdF-HFP) coating prepared by PEG-assisted phase separation.

**Figure S8**. Water contact angle results for HPC samples at the initial and after a 100-day cycle.

**Figure S9.** Characterization of HPC and other radiative cooling coupled with hydrogel methods.

**Table S4.** Structural Comparison of HPC and other radiative cooling coupled with hydrogel materials

| **Sample name** | **Material** | **Combination Method** | **Reference** |
| --- | --- | --- | --- |
| **P(VdF-HFP)@PAAm/alginate-CaCl_2_ bilayer** | P(VdF-HFP), PAAm, alginate, CaCl2 | Assemble toghter | ^[8]^ |
| **hydrogel-elastomer hybrids** | PAm, PBN, B-PDMS | Assemble toghter and wrapped | ^[12]^ |
| **TRE cooler** | CA fibrous, PVA-CaCl_2_ | Assemble toghter | ^[13]^ |
| **HPC** | P(VdF-HFP)@PAm-MF | Mechanical interlock | This work |

**Figure S10.** Stress-strain curves for pure PAm, initial HPC and after a 100-day cycle samples.

**Figure S11.** FDTD simulation of porous P(VdF-HFP) for different diameter.

**Figure S12.** Solar reflectance and emissivity spectra of HPC samples at initial and after a 100-day cycle test.

**Figure S13.** DSC curves of different HPC samples after LiCl solution immersion immediately.

**Figure S14.** Mass change plots of pure PAm and HPC sample without absorbed LiCl heated on an 80°C constant-temperature hotplate.

**Figure S15.** Mass percentage of each component of HPC samples with different concentration of LiCl solution after freshly immersed.

**Figure S16.** TGA curves of (a) HPC samples and (b) LiCl.

**Figure S17.** Specific heat capacity curves of different HPC samples at 20–100°C.

**Figure S18.** Dynamic water sorption curves of different HPC samples at 30% (a), 60% (b), and 90% (c) RH. All samples measure 10 × 10 × 5 mm.

**Figure S19.** Dynamic water absorption curves for HPC_45_ samples at the initial state and after 100 cycles, measured at 90±3% RH and 25±0.5 °C.

After a 100-day outdoor cycle, the water uptake capacity of the HPC sample at 90% RH and 25 °C decreased slightly from 3.90 to 3.53 g g⁻¹. The test results indicate that the HPC sample continues to exhibit fairly good water uptake capacity under continuous outdoor cycling conditions.

**Figure S20.** solar reflectance and infrared emittance spectra of the bare heater.

**Figure S21.** Outdoor images of test setup for different coolers.

**Figure S22.** Solar irradiance and humidity during the outdoor 30-minute with 2000 W m^-2^ thermal-shock on July 2, 2025. (corresponding to **Figure 4a** and **4b**)

**Figure S23**. Temperature variations (a) and temperature difference (b) of different coolers (bare heater, TPC, hydrogel, and HPC) under 2000 W m^-2^ thermal-shock and solar radiation with 30 minutes duration. (c) Solar irradiance and humidity during the outdoor 30-minute with 2000 W m^-2^ thermal-shock on July 12, 2026.

To further elucidate the cooling mechanism of the HPC, comparative experiments were conducted under identical outdoor solar radiation and thermal shock conditions, measuring the temperature profiles of a bare heater, a heater integrated with a traditional passive cooler (TPC), a heater integrated with a pure PAm hydrogel, and a heater integrated with the HPC. As shown in **Figure S23**, during a 30-minute outdoor thermal shock, the TPC, pure hydrogel, and HPC achieved average temperature reductions of −1.8 °C, 20.7 °C, and 27.2 °C relative to the bare heater, respectively. The TPC failed to effectively lower the heater temperature through radiation and convection due to its limited cooling power; instead, it suppressed natural convective dissipation and caused heat accumulation, maintaining the heater at a relatively high temperature. The pure PAm hydrogel provided substantial cooling through sensible-heat absorption and latent-heat evaporation during the thermal shock. However, lacking solar reflectance, it could not deliver effective sub-ambient cooling to the heater under non-shock conditions. Consequently, the pure hydrogel exhibited an overall average cooling performance 6.5 °C inferior to that of the HPC. This performance gap quantitatively demonstrates the cooling gain achieved by the HPC through synergistically combining radiative cooling with the sensible-heat absorption and latent-heat evaporation of the hydrogel, while also underscoring the necessity of radiative cooling for enabling sub-ambient temperature reduction in outdoor electronics.

**Figure S24.** Models for COMSOL simulation of solar radiation and thermal-shock. (a) Bare heater; (b) Heater combined with TPC; (c) Heater combined with HPC.

**Figure S25.** Curves of ambient emissivity and the coolers (TPC and HPC) emissivity that were established in the COMSOL simulation.

**Figure S26.** COMSOL simulation temperature variations (a) and temperature difference (b) of different cooling models under 2000 W m^-2^ thermal shock from 10:00 to 10:30.

**Figure S27.** Solar irradiance and humidity during an 8-interval, 30-minute-period of 2000 W m⁻² thermal shock process on July 4, 2025. (corresponding to **Figure 4e**)

**Figure S28.** Temperature difference between different coolers and the bare heater during an 8-interval, 30-minute-period of 2000 W m⁻² thermal shock process from 12:00 PM to 8:00 PM.

**Figure S29.** (a) Ambient temperature and humidity curves during different thermal shock power processes. (b) Temperature difference curves between the bare heater and the heater combined with HPC under different power thermal shocks.

**Figure S30.** Solar irradiance and humidity during the outdoor one hour with 2000 W m^-2^ thermal-shock on July 17, 2025. (corresponding to **Figure 4g** and **4h**)

**Figure S31.** Cross-sectional schematic diagram of different-sized HPC samples combined with heater (corresponding to **Figure 5a**).

**Figure S32.** Solar irradiance and humidity during the outdoor one hour with 2000 W m^-2^ thermal-shock on August 1, 2025 (Corresponding to **Figure 5b** and **5c**).

**Figure S33.** Temperature variations (b) and temperature difference (c) of different-area HPC under 2000 W m^-2^ thermal-shock with one hour duration indoors.

**Figure S34.** Models for COMSOL simulation of solar radiation and thermal-shock. (a) Heater combined with HPC-A_1_; (b) Heater combined with HPC-A_3_; (c) Heater combined with HPC-A_5_.

**Figure S35.** COMSOL simulation temperature variations (a) and temperature difference (b) of different HPC’s area under 2000 W m^-2^ thermal shock from 12:30 to 15:30.

**Figure S36.** Air ambient temperature and humidity curve of different-area HPC under 3-hour, 2000 W m^-2^ continuous thermal shock in outdoor environments over a 5-day cycle from September 1st to 5^th^, 2025 (Corresponding to **Figure 5d**).

**Figure S37.** Outdoor images of the setup for simulating the outdoor electronic with enclosures and heat source.

**Figure S38.** Solar irradiance and humidity during the outdoor three hour with 2000 W m^-2^ thermal-shock on September 11, 2025 (Corresponding to **Figure 5f** and **5g**).

**Figure S39.** Temperature and temperature difference variation curves from five consecutive days of outdoor solar radiation testing under different cooling strategies. November 22–26^th^, 2025.

**Figure S40.** Outdoor humidity and wind speed during the continuous outdoor testing process in **Figure S39**.

**Figure S41.** Temperature and temperature difference variation curves from five consecutive days of outdoor solar radiation testing under different cooling strategies. November 12–15^th^, 2025.

**Figure S42.** Outdoor humidity and wind speed during the continuous outdoor testing process in **Figure S41**.

**Figure S43.** COMSOL simulation temperature variations (a) and temperature difference (b) of different cooling model in one day.

**Figure S44.** Temperature and temperature difference variation curves for heater combined with different HPC areas during two consecutive days of outdoor testing. November 16–17^th^, 2025.

**Figure S45.** Outdoor humidity and wind speed during the continuous outdoor testing process in **Figure S44**.

**Reference**

[1]	J. Mandal, Y. Fu, A. C. Overvig, M. Jia, K. Sun, N. N. Shi, H. Zhou, X. Xiao, N. Yu, Y. Yang, *Science* **2018**, 362, 315.

[2]	A.-H. Cavusoglu, X. Chen, P. Gentine, O. Sahin, *Nature Communications* **2017**, 8.

[3]	D. L. McJannet, F. J. Cook, R. P. McGloin, H. A. McGowan, S. Burn, *Water Resources Research* **2011**, 47.

[4]	Q. Du, M. Yang, H. Sun, M. Li, J. Zhao, D. Li, D. Sun, *Advanced Functional Materials* **2025**, 35.

[5]	R. H. Galib, Y. Tian, Y. Lei, S. Dang, X. Li, A. Yudhanto, G. Lubineau, Q. Gan, *Nature Communications* **2023**, 14.

[6]	J. Fei, X. Zhang, D. Han, Y. Lei, F. Xie, K. Zhou, S.-W. Koh, J. Ge, H. Zhou, X. Wang, X. Wu, J.-Y. Tan, Y. Gu, Y. Long, Z. H. Koh, S. Wang, P. Du, T. Mi, B.-F. Ng, L. Cai, C. Feng, Q. Gan, H. Li, *Science* **2025**, 388, 1044.

[7]	J. Fei, D. Han, X. Zhang, K. Li, N. Lavielle, K. Zhou, X. Wang, J. Y. Tan, J. Zhong, M. P. Wan, E. Nefzaoui, T. Bourouina, S. Li, B. F. Ng, L. Cai, H. Li, *Nano Letters* **2023**, 24, 623.

[8]	J. Li, X. Wang, D. Liang, N. Xu, B. Zhu, W. Li, P. Yao, Y. Jiang, X. Min, Z. Huang, S. Zhu, S. Fan, J. Zhu, *Science Advances* **2022**, 8.

[9]	R. Wu, C. Sui, T.-H. Chen, Z. Zhou, Q. Li, G. Yan, Y. Han, J. Liang, P.-J. Hung, E. Luo, D. V. Talapin, P.-C. Hsu, *Science* **2024**, 384, 1203.

[10]	X. Zhao, T. Li, H. Xie, H. Liu, L. Wang, Y. Qu, S. C. Li, S. Liu, A. H. Brozena, Z. Yu, J. Srebric, L. Hu, *Science* **2023**, 382, 684.

[11]	K. Lin, S. Chen, Y. Zeng, T. C. Ho, Y. Zhu, X. Wang, F. Liu, B. Huang, C. Y.-H. Chao, Z. Wang, C. Y. Tso, *Science* **2023**, 382, 691.

[12]	L. Sun, D.-W. Sun, L. Xu, Y. Tian, R. Hu, Z. Zhu, *Chemical Engineering Journal* **2025**, 512, 162083.

[13]	Q. Xin, B. Ma, J. Ru, Y. Zhou, D. Jing, *Advanced Energy Materials* **2024**, 15.
